# Hepatocellular carcinoma patients with high circulating cytotoxic T cells and intra-tumoral immune signature benefit from pembrolizumab: results from a single-arm phase 2 trial

**DOI:** 10.1186/s13073-021-00995-8

**Published:** 2022-01-06

**Authors:** Jung Yong Hong, Hee Jin Cho, Jason K. Sa, Xiaoqiao Liu, Sang Yun Ha, Taehyang Lee, Hajung Kim, Wonseok Kang, Dong Hyun Sinn, Geum-Youn Gwak, Moon Seok Choi, Joon Hyeok Lee, Kwang Cheol Koh, Seung Woon Paik, Hee Chul Park, Tae Wook Kang, Hyunchul Rhim, Su Jin Lee, Razvan Cristescu, Jeeyun Lee, Yong Han Paik, Ho Yeong Lim

**Affiliations:** 1 Division of Hematology-Oncology, Department of Medicine, Samsung Medical Center, Sungkyunkwan University School of Medicine, Seoul, Republic of Korea; 2 Innovative Therapeutic Research Center, Precision Medicine Research Institute, Samsung Medical Center, Seoul, Republic of Korea; 3 Current address: Department of Biomedical Convergence Science and Technology, Kyungpook National University, Daegu, Republic of Korea; 4 Department of Biomedical Sciences, Korea University College of Medicine, Seoul, Republic of Korea; 5 Merck & Co., Inc., Kenilworth, NJ USA; 6 Department of Pathology and Translational Genomics, Samsung Medical Center, Sungkyunkwan University School of Medicine, Seoul, Republic of Korea; 7 Division of Gastroenterology, Department of Medicine, Samsung Medical Center, Sungkyunkwan University School of Medicine, Seoul, Republic of Korea; 8 Department of Radiation Oncology, Samsung Medical Center, Sungkyunkwan University School of Medicine, Seoul, Republic of Korea; 9 Department of Radiology and Center for Imaging Science, Samsung Medical Center, Sungkyunkwan University School of Medicine, Seoul, Republic of Korea; 10 Division of Hematology-Oncology, Department of Internal Medicine, Ewha Womans University College of Medicine, Seoul, Republic of Korea; 11 Department of Health Science and Technology, Samsung Advanced Institute for Health Science and Technology, Sungkyunkwan University, Seoul, Republic of Korea

**Keywords:** Carcinoma, Hepatocellular, Pembrolizumab, Biomarkers, Tumor

## Abstract

**Background:**

A limited number of studies have characterized genomic properties of hepatocellular carcinoma (HCC) patients in response to anti-PD-1 immunotherapy.

**Methods:**

Herein, we performed comprehensive molecular characterization of immediate (D-42 to D-1) pre-treatment tumor biopsy specimens from 60 patients with sorafenib-failed HCC in a single-arm prospective phase II trial of pembrolizumab. Objective response rate was the primary efficacy endpoint. We used whole-exome sequencing, RNA sequencing, and correlative analysis. In addition, we performed single-cell RNA sequencing using peripheral blood mononuclear cells.

**Results:**

The overall response rate of pembrolizumab in sorafenib-failed HCC patients was 10% ([6/60] *95% CI*, 2.4–17.6). In a univariate analysis using clinicopathological features, female gender, PD-L1 positivity, and low neutrophil-to-lymphocyte ratio (NLR) were identified as contributing factors to pembrolizumab response. Somatic mutations in CTNNB1 and genomic amplifications in MET were found only in non-responders. Transcriptional profiles through RNA sequencing identified that pembrolizumab responders demonstrated T cell receptor (TCR) signaling activation with expressions of MHC genes, indicating increased levels of T cell cytotoxicity. In single-cell sequencing from 10 pre- and post-treatment peripheral blood mononuclear cells (PBMCs), patients who achieved a partial response or stable disease exhibited immunological shifts toward cytotoxic CD8+ T cells. Conversely, patients with progressive disease showed an increased number of both CD14+ and CD16+ monocytes and activation of neutrophil-associated pathways.

**Conclusions:**

Taken together, HCC patients with infiltration of cytotoxic T cells, along with increased active circulating CD8+ T cells during pembrolizumab treatment and down-regulation of neutrophil-associated markers, significantly benefited from pembrolizumab treatment.

**Trial registration:**

NCT#03163992 (first posted: May 23, 2017)

**Supplementary Information:**

The online version contains supplementary material available at 10.1186/s13073-021-00995-8.

## Background

Hepatocellular carcinoma (HCC) accounts for the majority of primary liver malignancy and is the fourth leading cause of cancer-related mortalities worldwide [1, 2]. Asia constitutes disproportionately high numbers of HCC cases due to the endemic status of the hepatitis B virus (HBV). However, the rising incidence of HCC and associated mortality in Western countries are attributed by the increased number of cases with non-alcoholic fatty liver disease (NAFLD), metabolic syndrome, and obesity [1].

Immune checkpoint inhibitors for anti-programmed cell death 1 (PD-1), such as nivolumab and pembrolizumab, demonstrated durable clinical responses and favorable toxicity profiles in phase II clinical trials and were granted accelerated approval by the FDA for the second-line treatment of HCC (CheckMate 040 and KEYNOTE-224) [3, 4]. The overall response rates (*ORR*) in the CheckMate 040 trial (dose-expansion phase) and KEYNOTE-224 trials were 20% (*95% CI*, 15–26) and 17% (*95% CI*, 11–26), respectively [3, 4]. The identification of predictive and reliable biomarkers to predict the response to immune checkpoint inhibitors in HCC treatment remains a major challenge. Predictive biomarkers, such as microsatellite instability, tumor mutational burden, PD-L1 expression, and tumor-infiltrating T-cells, have been identified in other cancers in response to immunotherapy. Tumor mutational burden and T-cell-inflamed gene expression profile (GEP) have been shown to predict a favorable response to anti-PD-1 immunotherapy in several solid tumors [5–8]. However, there are a limited number of studies in exploring predictive markers for anti-PD(L)-1 treatment in HCC.

Hence, the primary objective in this study is to perform integrative genomics analyses of HCC patients who received pembrolizumab after sorafenib failure in hopes of facilitating identification of predictive biomarkers that can distinguish responders from non-responders. Pre-treatment tissue biopsies were obtained from the study participants immediately before pembrolizumab treatment and subjected to whole-exome sequencing (WES), RNA sequencing, and correlative analysis. In addition, we performed single-cell RNA sequencing (scRNA-seq) using peripheral blood mononuclear cells (PBMCs) that were derived from HCC patients before and after the pembrolizumab treatment in order to explore the dynamic cellular evolution of immune cells under pembrolizumab treatment.

## Methods

### Trial design and eligibility criteria

The study was a prospective, open-label, single-arm, phase 2 trial carried out at Samsung Medical Center, Seoul, Korea. A sample size of 55 patients was calculated according to the one-stage binomial design based on the null hypothesis of 5% overall response rate (*ORR*) and the alternative hypothesis of 18.5% *ORR* with 90% power and 5% one-sided alpha. Assuming about 10% attrition due to ineligibility and dropout, a total of 60 patients were recruited for this study. All statistical analyses were performed using R3.5.3.

The inclusion criteria for study participants were (1) to have histologically confirmed diagnosis of HCC (fibrolamellar and mixed hepatocellular/cholangiocarcinoma subtypes were not eligible); (2) to be ≥19 years of age; (3) to have an Eastern Cooperative Oncology Group performance status of 0 or 1; (4) to have Barcelona Clinic Liver Cancer (BCLC) Stage C disease, or BCLC Stage B disease not responsive to locoregional therapy or refractory to locoregional therapy, and not manageable by a curative treatment approach; (5) to have a Child-Pugh class A liver score; (6) with documented objective radiographic or clinical disease progression during first-line sorafenib therapy; (7) to have adequate organ function per protocol; (8) to have measurable disease based on RECIST1.1 criteria; (9) naive to anti-PD-1, anti-PD-L1, or anti-PD-L2 antibodies; (10) willing to provide fresh tissue for biomarker analysis, and based on the adequacy of the tissue sample quality, agree for assessment of biomarker status; (11) with a negative urine or serum pregnancy test within 72 h prior to receiving the first dose of study medication in female subject of childbearing potential; and (12) willing to use an adequate method of contraception per protocol in female and male subjects of childbearing potentials, for the course of the study through 120 days after the last dose of study medication. The exclusion criteria for study participants were (1) to participate and receive study therapy or has participated in a study of an investigational agent and received study therapy or used an investigational device within 4 weeks of the first dose of treatment; (2) to receive sorafenib within 14 days of first dose of study medication; (3) to have esophageal or gastric variceal bleeding within the last 6 months; (4) with a solid organ transplant; (5) with active autoimmune disease that has required systemic treatment in past 2 years (i.e., with the use of disease-modifying agents, corticosteroids, or immunosuppressive drugs); (6) with a diagnosis of immunodeficiency or is receiving systemic steroid therapy or any other form of immunosuppressive therapy within 7 days prior to the first dose of trial treatment; (7) to receive locoregional therapy to the liver (transcatheter chemoembolization [TACE], transcatheter embolization [TAE], radiation, radioembolization, or ablation), or major surgery to the liver or other sites within 6 weeks prior to the first dose of the study drug; (8) with a known additional malignancy that is progressing or requires active treatment. Exceptions include basal cell carcinoma of the skin or squamous cell carcinoma of the skin that has undergone potentially curative therapy or in situ cervical cancer, (9) with active central nervous system (CNS) metastases and/or carcinomatous meningitis. Subjects with previously treated brain metastases may participate provided they are stable (without evidence of progression by imaging for at least 4 weeks prior to the first dose of trial treatment and any neurologic symptoms have returned to baseline), have no evidence of new or enlarging brain metastases, and are not using steroids for at least 7 days prior to trial treatment, (10) with a known history of, or any evidence of, interstitial lung disease or active noninfectious pneumonitis; (11) with an active infection requiring systemic therapy; (12) with a history or current evidence of any condition, therapy, or laboratory abnormality that might confound the results of the trial, interfere with the subject’s participation for the full duration of the trial, or is not in the best interest of the subject to participate, in the opinion of the treating investigator; (13) has known psychiatric or substance abuse disorders that would interfere with cooperation with the requirements of the trial; (14) to be pregnant or breastfeeding, or expecting to conceive or father children within the projected duration of the trial, starting with the pre-screening or screening visit through 120 days after the last dose of trial treatment; (15) to receive prior therapy with an anti-PD-1, anti-PD-L1, or anti-PD-L2 agent; (16) with a known history of human immunodeficiency virus (HIV) (HIV 1/2 antibodies); (17) with an untreated active hepatitis B (e.g., HBsAg reactive) or hepatitis C (e.g., HCV RNA [qualitative] is detected); (18) to receive a live vaccine within 30 days of planned start of study therapy. All patients provided written informed consent before enrollment (ClinicalTrials.gov, NCT#03163992). The trial protocol was approved by the Institutional Review Board at Samsung Medical Center (Seoul, Korea) and was conducted by following the Declaration of Helsinki and Guidelines for Good Clinical Practice. The approved trial protocol and the revision history of the protocol can be checked in the additional files (Additional files 1 and  2).

Pembrolizumab 200 mg was administered as a 30-min intravenous infusion every 3 weeks until further disease progression or unacceptable toxicity was documented, or up to 24 months. Computed tomography (CT) scan and/or magnetic resonance imaging (MRI) were performed every two cycles to evaluate the tumor response according to the RECIST 1.1 criteria. As per the RECIST1.1, objective response rate (*ORR*) was considered at the primary efficacy endpoint. The response evaluation was performed every 2 cycles of pembrolizumab. The progression-free survival (*PFS*) was defined as the time from the start of treatment until the date of disease progression or death resulting from any cause. The overall survival was measured from the start of treatment to the date of death from any cause. The response rate was calculated as the percentage of patients experiencing a confirmed complete response (CR) or partial response (PR). Toxicities were defined and graded based on the National Cancer Institute Common Terminology Criteria for Adverse Events 4.0 [9].

### Sample collection

Tumor tissues were obtained using core-needle biopsy or excisional biopsy as clinically indicated before the initiation of pembrolizumab treatment. Only tissue samples within 6 weeks before initiation of treatment were allowed. If tumor content was estimated at more than 40% after a thorough pathological review, tumor DNA and RNA were extracted from tumor tissues using QIAamp Mini Kit (Qiagen) according to the manufacturer’s instructions; RNase A (Qiagen) was used during DNA extraction. The qualitative and quantitative analysis of the extracted DNA was performed with an ND1000 spectrophotometer (Nanodrop Technologies, ThermoFisher) and Qubit fluorometer (Life Technologies).

We also obtained peripheral blood samples from before and after the pembrolizumab treatment for 2 cycles. The peripheral mononuclear cells obtained were subjected to scRNA-seq using the 10x Genomics single-cell library.

### PD-L1 immunohistochemistry

Tumor tissue sections were freshly cut to 4 μm and mounted on microscope glass slides (Fisherbrand Superfrost Plus, ThermoFisher), then dried at 60 °C for 1 h. IHC staining was carried out on a Dako Autostainer Link 48 system (Agilent Technologies) using the Dako PD-L1 IHC 22C3 pharmDx kit (Agilent Technologies) with EnVision FLEX visualization system. Slides were counterstained with hematoxylin according to the manufacturer’s instructions. PD-L1 protein expression was determined using a combined positive score (*CPS*), calculated by the number of PD-L1 staining cells (tumor cells, lymphocytes, macrophages) divided by the total number of viable tumor cells, multiplied by 100. The specimens were considered to have PD-L1 expression if *CPS* ≥1.

### WES pipeline

WES reads were aligned to the reference human genome (GRCh37) using BWA-MEM [10] followed by preprocessing steps including duplicate marking, indel realignment, and base recalibration using the Genome Analysis Toolkit (GATK, v3.6 and v4.1.3) [11]. Resulting BAM files were used to obtain somatic single nucleotide variants/small insertion and deletions (SNV/INDELs) and copy number variations (CNVs) MuTect2 in GATK generated somatic SNV/INDEL calls by comparing BAM files from tumor and matched normal samples [12]. SNV/INDELs higher than 0.0000025 population allele fraction in gnomAD [13] were filtered out to remove possible germline mutations. Somatic mutations were annotated with variant effect predictors [14]. SNVs with mutant reads of equal or less than four in tumor samples were also eliminated. Tumor mutation burden (TMB) for a subject was defined as the number of somatic non-synonymous SNVs that passed all the filters described. The mutational signature analysis was performed using the deconstructSigs package (v1.8.0) in R, which selects combinations of known mutational signatures that account for the observed mutational profile in each sample [15]. WES-based CNV estimation was performed by ngCGH (python package).

### RNA-Seq pipeline

RNA-Seq reads were aligned by STAR (v2.6.1d) [16] and the gene expression levels were quantified by fragments per kilobase of transcript per million (FPKM) mapped reads. Log2-transformed FPKM values were used for further analyses except for differentially expressed gene (DEG) extraction. DEGs were extracted using DEseq2 [17] with read counts per gene which were generated by DEGseq [18]. Single-sample GSEA (ssGSEA) scores were used to estimate gene signature expression levels for REACTOME MET receptor activation and neutrophil markers [19] in a single sample using R package GSVA [20]. Geneset enrichment analysis between responders and non-responders was performed by GSEA-P [21].

### Single-cell RNA-sequencing data process and analysis

Single-cell RNA-sequencing reads were aligned to the GRCh38 human genome reference and quantified using cellranger (version 3.1.0). Further analyses were conducted using Seurat (version 3.1.4). Unique feature or gene counts that were greater than 3000 or less than 200 were excluded to account for potential doublets, multiplets, low-quality cells, or empty droplets. Afterwards, cells with greater than 10% mitochondrial genome content were further excluded from the analysis. As a result, a total of 26,541 cells passed the QC filters. Raw feature counts were then log-normalized and scaled and subjected to linear dimensional reduction using principal component analysis (PCA). Cell clusters were then identified using the *K*-nearest neighbor (KNN) graph model based on the Euclidean distance in PCA space and *t-*stochastic neighbor embedding (tSNE) algorithm for visualization. Different cell type clusters were identified through performing differentially expressed gene analysis for each cluster and annotated based on the expression of representative markers.

## Results

### Patient characteristics

The baseline characteristics of the study participants are summarized in Table 1. All 60 patients were pathologically confirmed as HCC and received sorafenib as first-line systemic treatment but showed tumor progression prior to the enrollment of this study. The first patient enrollment date was 26 Dec 2017 and the last patient enrollment date was 11 Jun 2019. Forty-eight patients (80.0%) received pembrolizumab as the second-line treatment, while 12 patients (20.0%) received it as a third- or greater-line systemic treatment. Forty-eight (80.0%) were male, and the median age was 60 (range, 37–84). There were 46 patients (76.7%) with HBV infection, 6 (10.0%) with hepatitis C virus (HCV) infection, 1 (1.7%) with alcoholic cirrhosis, 1 (1.7%) with primary biliary cirrhosis, and 6 (10.0%) with unknown etiology. Alpha-fetoprotein (AFP) levels of 30 patients (50%) were higher than 400 ng/ml at baseline, and 52 (86.7%) showed extrahepatic metastasis.

**Table 1:** Baseline characteristic of patients

		***n*** = 60 (%)
**Age**	< 60	28 (46.7%)
	≥ 60	32 (53.3%)
**Sex**	Female	12 (20.0%)
	Male	48 (80.0%)
**ECOG performance status**	0	9 (15.0%)
	1	51 (85.0%)
**Pathologic confirmation**	No	0 (0.0%)
	Yes	60 (100%)
**Child-Pugh classification**	A5	41 (68.3%)
	A6	19 (31.7%)
**Etiology**	Hepatitis B	46 (76.7%)
	Hepatitis C	6 (10.0%)
	Alcohol	1 (1.7%)
	Primary biliary cirrhosis	1 (1.7%)
	Others	6 (10.0%)
**Alpha-fetoprotein**	≥ 400	30 (50.0%)
	< 400	30 (50.0%)
**Extrahepatic disease**	No	8 (13.3%)
	Yes	52 (86.7%)
**Prior treatment**	Transplantation	0 (0.0%)
	Surgery	17 (28.3%)
	Locoregional therapy (RFA, TACE)	41 (68.3%)
	Radiotherapy	31 (51.7%)
**Prior chemotherapy**	1	48 (80.0%)
	≥ 2	12 (20.0%)
**Prior sorafenib**	No	0 (0.0%)
	Yes	60 (100%)

*ECOG*, Eastern Cooperative Oncology Group; *RFA*, radiofrequency ablation; *TACE*, transarterial chemoembolization

### Clinicopathological profiles and response to pembrolizumab

**Fig. 1:**
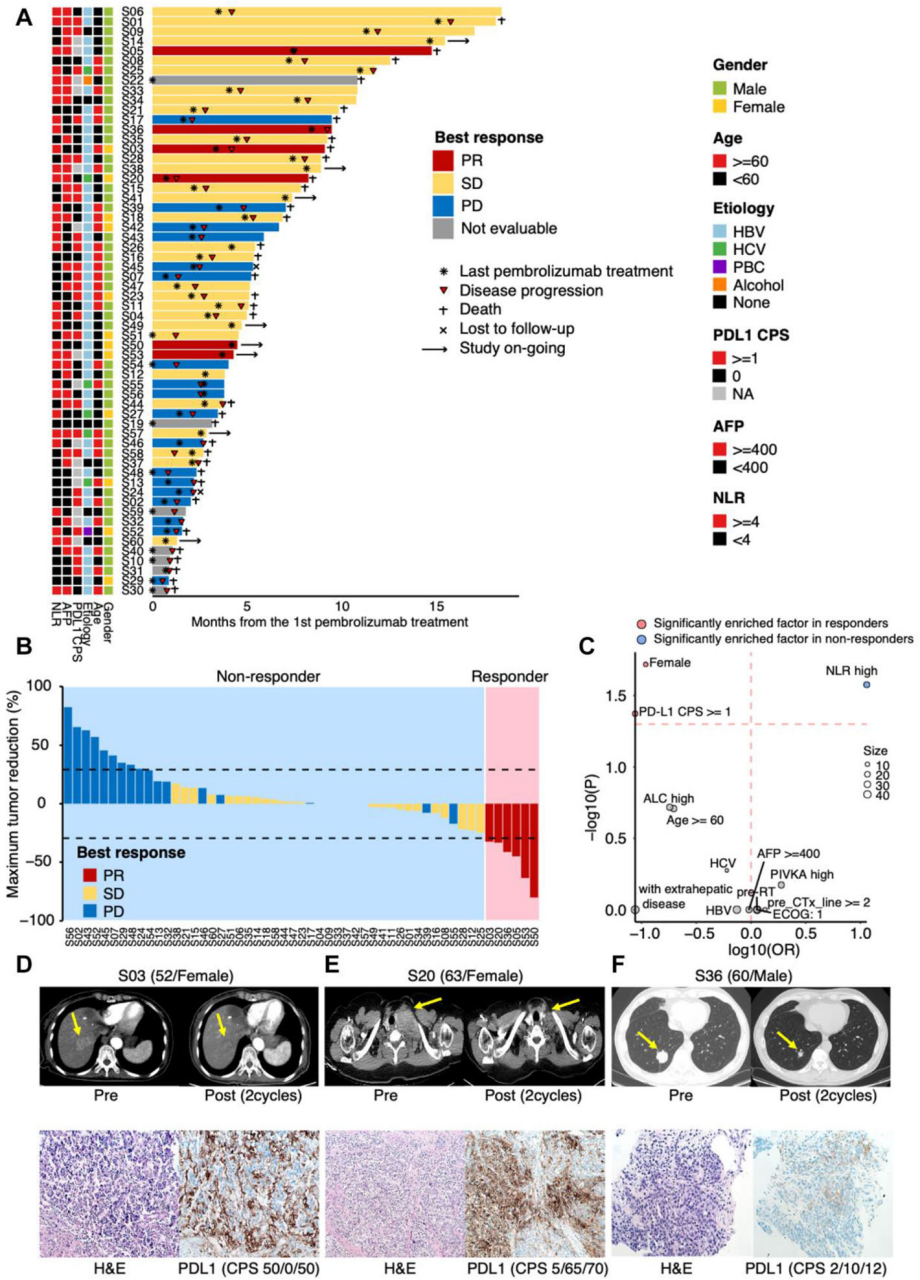
Clinicopathological features in association with response to pembrolizumab. S represents each patient’s identification number. **A** Swimmer plot for enrolled patients (*n =* 60). Each lane represents a single patient’s data. **B** Waterfall plot of response to pembrolizumab. The *Y*-axis represents the percentage of maximum tumor reduction assessed according to RECIST 1.1 criteria. The lower dotted line represents tumor reduction of 30% per RECIST, which defines PR (responder). Patients with bars in blue (PD) and yellow (SD) were determined to be non-responders. **C** Univariate analysis to identify clinicopathological features associated with response to pembrolizumab. Odds ratio and *p* value for each feature were computed via Fisher’s exact test. The size of the circle represents the number of samples. **D**–**F** Tumor reduction after pembrolizumab treatment in three responders who have available PD-L1 status by immunohistochemistry (IHC) (upper panel). H&E staining and PD-L1 positivity for the three responders (lower panel). PR, partial response; SD, stable disease; PD, progressive disease; *CPS*, combined positive score, AFP, alpha-fetoprotein; NLR, neutrophil-to-lymphocyte ratio

As of July 20, 2019, the median follow-up duration was 5.1 (range 0.8–18.5) and the median number of treatment cycles completed was four (range, 1–22) (Fig. 1A and Additional file 3: Table S1). The percentage of maximum tumor reduction after pembrolizumab treatment for each patient was assessed according to RECIST 1.1 criteria (Fig. 1B). An overall response rate (*ORR*) was 10.0% (0 complete response (CR), 6 partial responses (PRs) (6/60 [*95% CI*, 2.4–17.6])), 50% of patients achieved stable disease (SD) (30/60, 50% [*95% CI*, 18.4–41.6]), and 28.3% of patients had progressive disease (PD) (17/60, 28.3% [*95% CI*, 7.5–26.5]). Seven patients (11.7%) were not assessed due to loss of follow-up (Fig. 1A, B). When we evaluated the association between response to pembrolizumab and clinicopathological profiles, patients with low neutrophil-to-lymphocyte ratio (NLR) (*p* = 0.027), positive PD-L1 expression (combined positive score (*CPS*) ≥ 1) (*p* = 0.042), and female patients (*p* = 0.019) were significantly prevalent in pembrolizumab responders (Fig. 1C and Additional file 4: Table S2). When we evaluated the association between disease control (PR + SD) to pembrolizumab and clinicopathological profiles, low AFP (*p* = 0.002) and low PIVKA-II (*p* = 0.038) were significantly prevalent in patients who achieve disease control from pembrolizumab (Additional file 4: Table S2). Toxicities were manageable and safety profiles were demonstrated in Additional file 4: Table S3. Representative CT scans and microscopic findings of patients (H&E staining and PD-L1 expression) who achieved PR to pembrolizumab are illustrated in Fig. 1D–F.

The median progression-free survival (*PFS*) and overall survival (*OS*) of the entire study cohort (*n =* 60) were 2.8 months (*95% CI*, 2.6–4.7) and 8.3 months (*95% CI*, 5.4–10.9), respectively (Additional file 4: Fig. S1A and B). Survival analyses between patients with pembrolizumab-refractory HCC and patients who achieved PR or SD to pembrolizumab are described in Additional file 4: Fig. S1C-F.

### Genomic landscape and molecular profiles associated with response to pembrolizumab

**Fig. 2:**
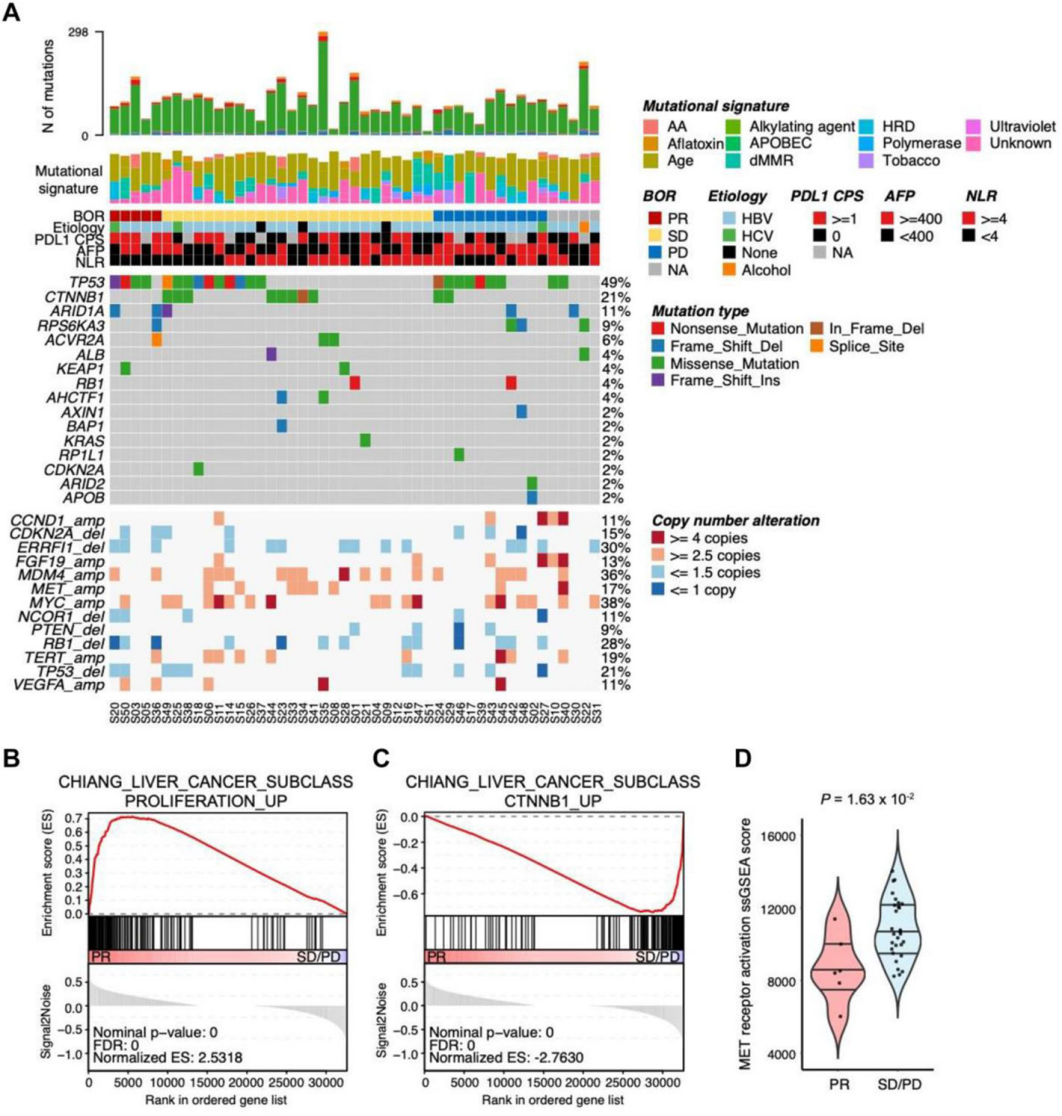
The genomic landscape and clinicopathological features. S represents each patient’s identification number. **A** Top panel shows the number of somatic mutations of each sample, mutation signature (COSMIC version 2), BOR, etiology, the scores of PD-L1 *CPS*, AFP, and NLR. The middle panel shows the mutational landscape of non-synonymous mutation for frequently altered genes in HCC. Green, red, purple, blue, brown, orange, and gray tiles indicate missense, nonsense, frameshift-insertion, frameshift-deletion, inframe deletion, splice site mutation, and wild-type. The bottom panel displayed copy number alteration for frequently amplified/deleted genes in HCC. Tiles in red, salmon, sky blue, and blue indicate amplification (copy number (*CN*) ≥ 4), gain (*CN* ≥ 2.5), loss (*CN* ≤ 1.5), and deletion (*CN* ≤ 1), respectively. **B** GSEA plot representing CHIANG_LIVER_CANCER_SUBCLASS_PROLIFERATION_UP pathway was significantly enriched in responders (PR) (*FDR* = 0 and normalized enrichment score (*NES*) = 2.53). **C** GSEA plot representing CHIANG_LIVER_CANCER_SUBCLASS_CTNNB1_UP pathway was significantly enriched in non-responders (SD/PD) (*FDR* = 0 and *NES* = −2.76). **D** ssGSEA scores of Reactome MET receptor activation geneset were significantly higher in non-responders (SD/PD) than responders (PR) (Wilcoxon rank-sum *p*-value = 0.016). ssGSEA scores were calculated using GSVA package in R. BOR, best overall response; *CPS*, combined positive score; AFP, alpha-fetoprotein; NLR, neutrophil-to-lymphocyte ratio; PR, partial response; SD, stable disease; PD, progressive disease

To identify genomic and transcriptomic predictors for pembrolizumab in HCC, we performed whole-exome sequencing (WES) and RNA sequencing on 47 (responders, *n =* 5; non-responders, *n =* 37; not evaluable, *n =* 5) and 40 pre-treatment HCC samples (responders, *n =* 6; non-responders, *n =* 29; not evaluable, *n =* 5), respectively. Based on WES data, a similar prevalence of non-synonymous mutations was found in *TP53*, *CTNNB1*, and *ARID1A*, as reported by other HCC genomic studies (Fig. 2A) [1, 22]. We did not observe any mutual exclusivity pattern between *CTNNB1* mutations (*n =* 10, 21.3%) and *TP53* mutations (*n =* 20, 48.9%) (odds ratio = 1.05) [23]. Although there were no significant genomic factors associated with response to pembrolizumab due to the small sample size of responders, interestingly, all patients with *CTNNB1* mutations were non-responders to pembrolizumab (Additional file 5: Table S4). The samples with *CTNNB1* mutations showed up-regulated CTNNB1-HCC class pathway compared with *CTNNB1* wild-type tumor samples, suggesting that the discovered CTNNB1 mutations in this cohort induced the activation of WNT/β-catenin pathway (Additional file 4: Fig. S5C and D). Consistent with *CTNNB1* mutational status, CTNNB1-HCC subclass pathway was enriched in non-responders while responders showed up-regulated proliferation-HCC subclass pathway according to geneset enrichment analysis (GSEA) analysis [24] (Fig. 2B, C). Furthermore, we have pathologically evaluated the prevalence of tumor-infiltrating lymphocytes (TILs) and PD-L1 expression level based on *CTNNB1* somatic mutation. There were no statistically significant differences in terms of both TILs and PD-L1 expression according to *CTNNB1* somatic mutation (Additional file 4: Fig. S2A and B). Mutational status of *TP53* or *ARID1A* was not significantly associated with response to pembrolizumab in HCC (*p* = 0.34 and 0.063, respectively), but mutations in *TP53* and *ARID1A* genes were more frequent in responders than non-responders (odd ratios = 4.55 and 10.4, respectively). The prevalence of tumor mutation burden (TMB)-high at the clinically validated cutoff of 175 missense mutations/exome was very low (2.4% [1/42]), and high TMB could not be evaluated for a potential predictive biomarker of pembrolizumab in this study (Additional file 4: Fig. S3) [7, 25]. We also estimated copy number variations in the genes which were previously reported as frequently amplified or deleted genes (Fig. 2A). Although there were no significant associations between responses to pembrolizumab and gene copy number alterations, we found that copy gains in *MET* were unique to the non-responder group (0/5 vs. 7/37), and accordingly, non-responders exhibited increased expression levels for MET receptor activation markers compared to responders (Fig. 2A, D).

**Fig. 3:**
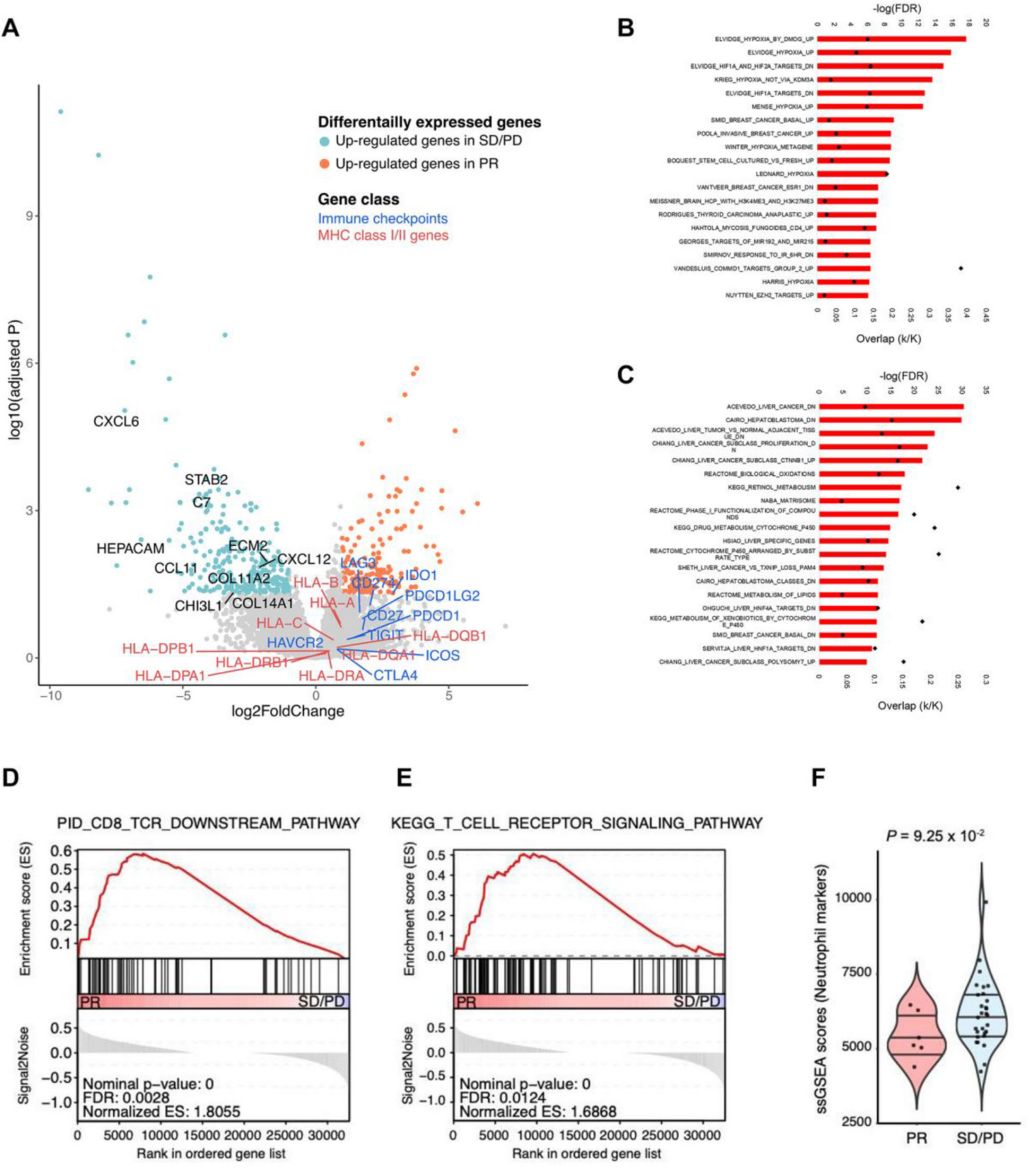
Gene expression profiles in association with response to pembrolizumab. **A** A volcano plot showing the differentially expressed genes (DEGs) between responders and non-responders. Red and green dots indicate the up-regulated genes in responders (adjusted *p*-value < 0.05 and log2 fold change > 1) and the up-regulated genes in non-responders (adjusted *p*-value < 0.05 and log2 fold change < − 1). The *X*-axis represents the log2-fold changes in expression levels, and the *Y*-axis represents the statistical significances (-log10-adjusted *p*-value) between responders and non-responders. Each dot represents one gene. **B**, **C** A barplot illustrating the top 20 significantly overlapped MSigDB gene sets (*FDR* < 0.05) with the DEGs generated in **A**. Hypoxia was associated with up-regulated genes in responders (**B**), and liver/HCC-associated genesets were overlapped with up-regulated genes in non-responders (**C**). Red bar represents statistical significances (-log10 scale) and black dot indicates the proportion of overlapped genes in genesets. **D**, **E** GSEA plots representing PID_CD8_TCR_DOWNSTREAM pathway (*FDR* = 0.0028, *NES* = 1.81) (**D**) and KEGG_T_CELL_RECEPTOR_SIGNALING pathway (*FDR* = 0.012, *NES* = 1.69) (**E**) were significantly enriched in responders (PR). **F** ssGSEA scores of gene markers for neutrophil were up-regulated in non-responders (SD/PD) (Wilcoxon rank-sum *p* value = 0.093). DEG, differentially expressed gene; *FDR*, false discovery rate; GSEA, geneset enrichment analysis; *NES*, normalized enrichment score; PR, partial response; SD, stable disease; PD, progressive disease

Next, we analyzed immunological features of pembrolizumab responders using 40 RNA-sequencing data. First, DESeq2 identified differentially expressed genes (DEGs) between responders and non-responders (log2FC > 1 and adjusted *p* < 0.05) (Fig. 3A). To biologically characterize the DEGs, we examined which molecular pathways were overlapped with DEGs using MSigDB curated gene sets (C2) [26] and revealed that hypoxia-associated genesets were enriched in up-regulated genes in responders (Fig. 3B). Previous studies demonstrated that hypoxia conditions enhanced effector function of CD8 T cells and cytotoxicity of cytotoxic T lymphocytes (CTLs) [27, 28]. GSEA showed that T cell receptor (TCR) signaling pathways were significantly enriched in responders, and also, immune checkpoints and MHC genes showed higher expression levels in responders compared to non-responders (Fig. 3A, D, E). In sum, studies using mRNA expression profiles demonstrated that increased cytotoxic T cell function under hypoxia circumstances in the tumor microenvironment of responders results in more powerful PD-l blockade effects on sorafenib-failed HCC patients. On the other hand, liver- and HCC-specific genes were up-regulated in non-responders, and extracellular matrix (ECM)-associated genes were also included in non-responder DEGs (Fig. 3A, C). According to clinical-pathological profiles, low NLR was one of the prognostic markers (Fig. 1C). We evaluated whether neutrophil markers were transcriptomically down-regulated in responders and confirmed that neutrophil gene markers were relatively lower expressed in responders compared to non-responders (Fig. 3F). The NLR showed the significant positive correlation with the expression of neutrophil gene markers (*p* = 0.037). Also, the patients with high-NLR had the higher expression levels of neutrophil gene markers than the patients with low NLR (Wilcoxon rank-sum *p*-value = 0.039) (Additional file 4: Fig. S4A and B). An integrated analysis of the molecular features showed that the 6 responders to pembrolizumab in our study were characterized by lower levels of immunosuppressive tumor endogenous or microenvironment elements (*CTNNB1* mutations, neutrophils, ECM, and stroma signatures), and increased levels of T-cell cytotoxicity and cell proliferation, delineating a particular class of HCC that is most likely to benefit from pembrolizumab.

### Single-cell RNA sequencing reveals cellular composition and immunological changes in response to pembrolizumab

**Fig. 4:**
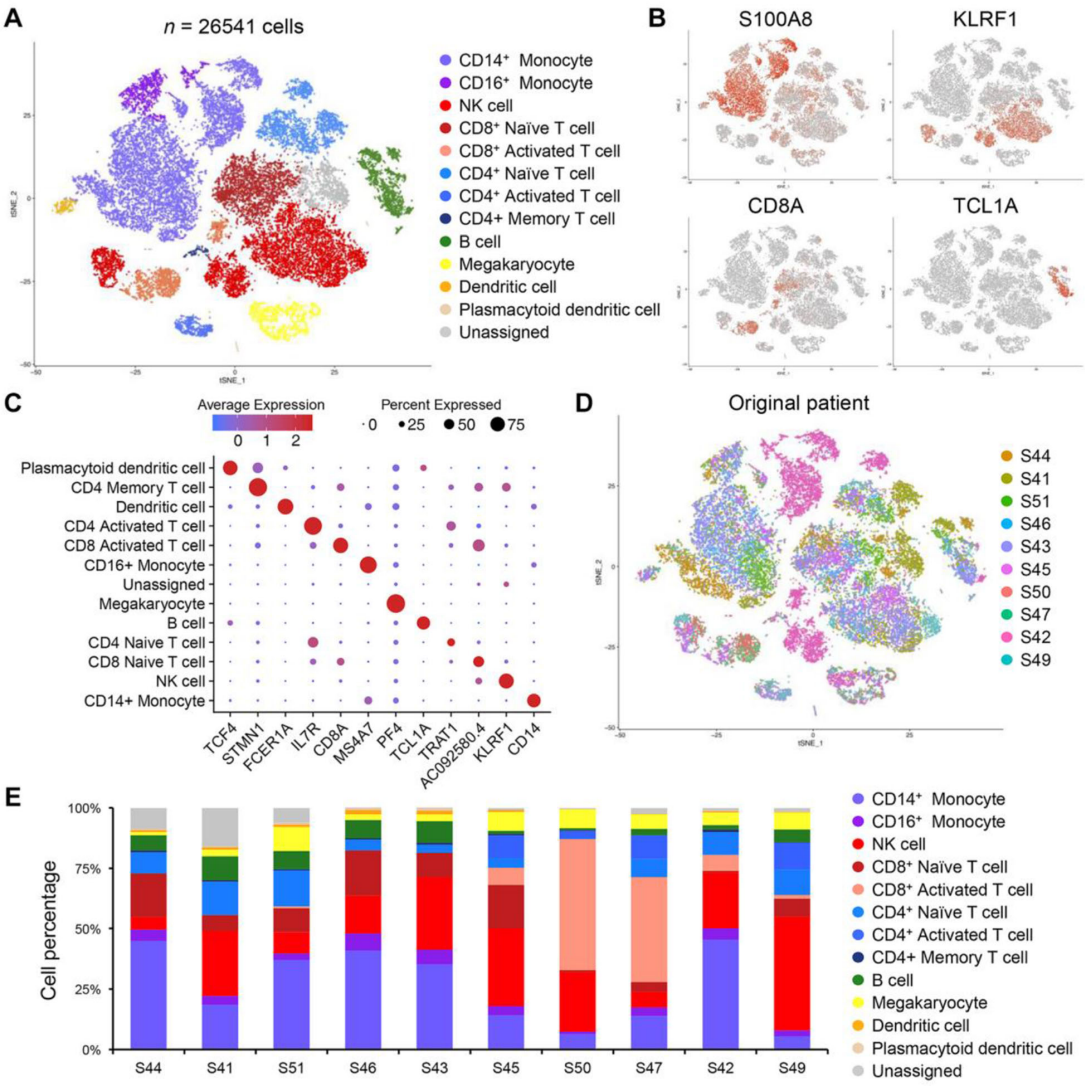
Single-cell characterization of immune cells via scRNA-seq. **A** t-stochastic neighbor embedding (tSNE) analysis of 26,541 immune cells from 10 patients before and after 2 cycles of pembrolizumab treatment. Cell type clusters were identified and annotated using scCATCH and are denoted by distinct color based on respective cell types. **B** tSNE analysis of immune cells colored by representative cell type markers, including *S100AB*, *KLRF1*, *CD8A*, and *TCL1A*. **C** Dot plot analysis of each cell type cluster and their representative markers. Colors indicate average expression levels, while the size of each dot represents the percentage of cells expressing the respective marker. **D** tSNE analysis of immune cells colored by patient identity. **E** Bar graph representation of immune cell composition based on individual patient. Each immune cell type has been normalized based on individual patient

Single-cell RNA sequencing (scRNA-seq) provides innovative opportunities to dissect and delineate complex cellular hierarchy at single cell resolution. To decipher the complexity of cellular diversity and evolutionary trajectory in immune cell composition in response to pembrolizumab, we obtained peripheral blood mononuclear cells from before and after the pembrolizumab treatment. Ten paired pre-treatment (baseline) and post-treatment (6-week pembrolizumab) samples of PMBC from ten patients with BLCL stage C disease were subjected to scRNA-seq using the 10x Genomics single-cell library. A total of 54,017 single cells were detected with an average of 2700 cells per case, and after the alignment on the hg19 reference genome, 32,738 features or unique genes were detected. Various quality-controlled (QC) criteria were implemented to account for potential doublets and mitochondrial gene content. Overall, 26,541 single cells and 18,221 unique genes passed the QC filter. Dimensional reduction analysis via *t-*distributed stochastic neighbor embedding (tSNE) revealed 18 distinct cell type clusters that are enriched for specific immune cell populations, including five myeloid clusters, seven T cell clusters, one B cell cluster, and three natural killer (NK) cell clusters (Fig. 4A). Each individual cellular compartment demonstrated uniquely expressed transcriptomes, including *S100A8* for CD14+ monocytes, *KLRF1* for NK cells, *CD8A* for CD8+ activated T cells, *TCL1A* for B cells, *PF4* for megakaryocytes, etc. (Fig. 4B, C). We discovered that this heterogeneous mixture of immune cell populations clustered together regardless of patient origin, suggesting consistency of immune cell types across individual patients (Fig. 4D). Interestingly, each patient manifested distinct immune cell distributions, some demonstrating enrichments of activated cytotoxic CD8+ T cells (S50, S47) and CD4+ naïve T cells (S41), while others were marked by notable accumulation of NK cells (S43, S45, S42, S49) and CD14+/CD16+ monocytes (S44, S46, S42) (Fig. 4E). We suspect that such dynamic cellular architecture within peripheral blood potentially contributes to overall pembrolizumab response within a clinical framework.

**Fig. 5:**
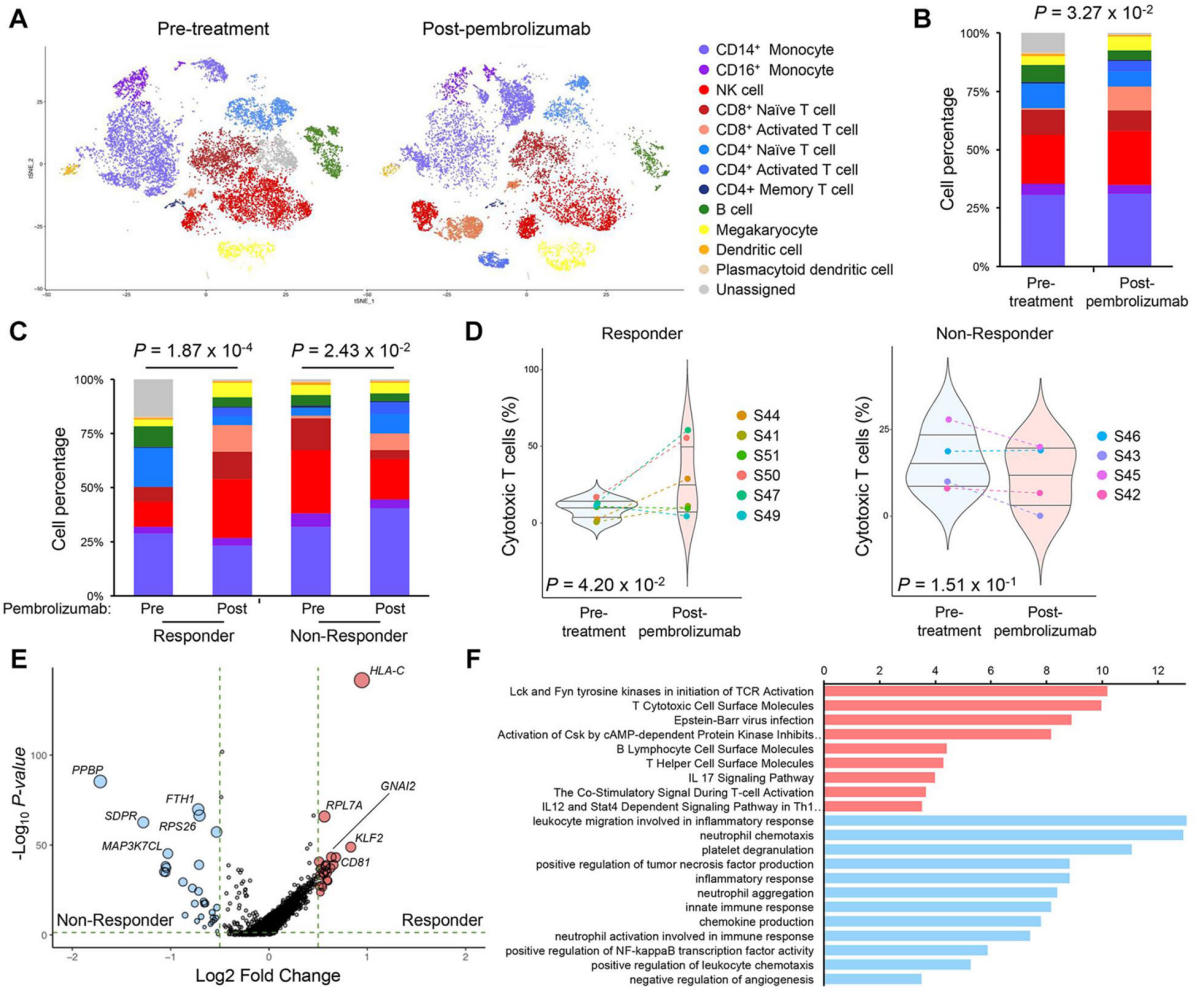
Immunological changes of immune cells in response to pembrolizumab. **A** tSNE analysis of immune cells colored by inferred cell type and separated by pembrolizumab treatment time point. Pre-treatment (left panel) and post-treatment (right panel). **B** Bar graph representation of immune cell composition based on pembrolizumab treatment time point. The *p*-value has been calculated using the chi-squared test. **C** Bar graph representation of immune cell composition based on pembrolizumab treatment time point and response. The *p*-values have been calculated using chi-squared tests. **D** Violin plot representation of cytotoxic T cells between pre- and post-pembrolizumab treatment in patients who achieve PR or SD (left panel) and patients with PD (right panel). The *p* values are calculated by a two-sided Wilcoxon rank-sum test. **E** Volcano plot representation of differentially expressed gene analysis in cytotoxic T cells between patients who achieve PR or SD and patients with PD. Genes with > 0.5 or < − 0.5 log_2_ fold change and < 0.05 *p-*value are colored in red and blue, respectively. **F** Gene Ontology (GO) analysis of differentially expressed genes in **E**. tSNE, *t-*stochastic neighbor embedding; PR, partial response; SD, stable disease; PD, progressive disease

We next focused on immunological changes in response to checkpoint blockade. When we differentiated immune cell type clusters based on pre- and post-treatment time points, we observed an overall increased frequency of activated CD4+ and CD8+ T cells and reduction of both CD4+ and CD8+ naïve T cells, further advocating the notion that immune checkpoint therapy primarily affects CD8+ T cells [29–31] (Fig. 5A, B). We further categorized immune cell populations based on clinical response to pembrolizumab and discovered that such immunological shifts toward cytotoxic CD8+ T cell were more prevalent in patients who achieve PR or SD (Fig. 5C). Notably, we also found that patients with PD demonstrated an increased number of both CD14+ and CD16+ monocytes, consistent with previous studies that have shown potential association between infiltration of monocytes with impediment of natural T cell functions [32–34]. Overall increased frequency of cytotoxic T cells was significantly more evident in the patients who achieve PR or SD to pembrolizumab (Fig. 5D). To elucidate global transcriptional regulatory networks that govern cytotoxic T cells in clinical response to pembrolizumab, we performed genome-wide differentially expressed gene analysis. Notably, cytotoxic T cells that were derived from the patients who achieved PR or SD exhibited initiation of T-cell receptor activation via Lck and Fyn tyrosine kinases. Conversely, patients with PD mainly showed enrichments of molecules that were associated with neutrophil activation. We suspect that potential interaction between cytotoxic T cells with neutrophils in T cell-inflamed microenvironment could contribute to the acquisition of immunosuppressive properties. Collectively, scRNA-seq revealed overall immunological shifts of cytotoxic T cells that potentially attribute the overall clinical benefit of pembrolizumab in HCC patients.

## Discussion

Clinical trials, such as CheckMate 459, KEYNOTE-240, and IMbrave150, have demonstrated that a substantial proportion of HCC patients significantly benefitted from anti-PD-1 treatment [35–37], and clinical application of anti-PD-1 immunotherapy in HCC is expected to widen in the future. To the best of our knowledge, this is the first study to perform comprehensive molecular characterization of HCC patients in response to pembrolizumab treatment following progression on sorafenib in a phase II clinical trial. Somatic mutation in *CTNNB*1, accompanied by activation of CTNNB1 signaling pathway, was prevalent in non-responders. Furthermore, frequent genomic amplification of *MET* was also evident in non-responders. Conversely, through genome-wide transcriptome analysis, we observed activation of the TCR signaling pathway in responsive patients. Lastly, we demonstrated that HCC patients with increased highly activated circulating CD8+ T cells in their peripheral blood during pembrolizumab treatment achieved significant clinical benefit to pembrolizumab, while refractory patients demonstrated enrichments of CD14+/CD16+ monocytes and NK cells.

Previous studies have identified distinct molecular subtypes within HCC with different prognoses [1, 24, 38, 39]. Transcriptional analysis indicated that responders resembled transcriptomic features of proliferation class HCC while non-responders exhibited activation of CTNNB1 class HCC-like GEP. We suspect that non-proliferation class HCC presents a higher incidence of *CTNNB1* mutation [1, 40, 41], which promotes activation of the WNT/β-catenin pathway, resulting in immune evasion and resistance to anti–PD-1 immunotherapy [42]. Hence, HCC patients with *CTNNB1* somatic mutation and/or non-proliferation class remained less responsive to anti-PD-1 treatment. Since there is more recent evidence showing that activating WNT/β-catenin signaling is associated with innate resistance to immune checkpoint inhibitors, further research is needed in terms of *CTNNB1* mutation and subsequent activation of WNT/β-catenin signaling in association with immunotherapy resistance in HCC [23, 43].

GEP of HCC with cellular annotation of matched PBMCs at pre- and post-treatment of pembrolizumab via scRNA-seq revealed that enhanced cytotoxic T cells constitute a major immunological niche within the tumor microenvironment (TME) in responders. On the other hand, immunosuppressive TME was evident in non-responders during anti-PD-1 treatment course, characterized by an increased number of CD14+/CD16+ monocytes and enrichment of neutrophil-activation markers. Consistently, low NLR was a significant predictor of response to pembrolizumab based on clinicopathological factor analysis. A previous study reported that neutrophils could acquire immunosuppressive characteristics in T cell-inflamed cancers, and the efficacy of cancer immunotherapies was improved via c-MET inhibition by impairing neutrophil mobilization and recruitment into tumors [44]. Besides, aberrant c-Met activity has been implicated in the development of HCC, and c-Met is still a therapeutically relevant target in HCC [45, 46]. In this study, genomic profiles revealed that *MET* copy number gain was unique to non-responders, and this suggests that consolidating co-inhibition of PD-1 with MET could enhance the clinical benefit of immunotherapies in HCC patients. Additionally, recent studies have shown empirical evidences on the role of pro-tumorigenic monocytes and macrophages during immunotherapy through modulation of immune-suppressive environment, subsequently inhibiting T cell recruitment and functions [47–49]. They present specific cell-surface antigens that are therapeutically exploitable and can be potentially employed synergistically with immune checkpoint inhibitors [50].

Screening of PD-L1 expression via immunohistochemical analysis is considered a cost-effective and efficient tool for selecting the potential candidates who may benefit from anti-PD-1 therapy across several tumor types [51]. A recent report showed that PD-L1 expression could predict the overall response to pembrolizumab up to 20 different cancer types [52]. However, clinical application of PD-L1 expression, measured through IHC staining has remained controversial in patients with advanced HCC [3, 4]. Interestingly, our results confirmed that high expression of PD-L1 score (*CPS* ≥ 1) significantly predicted overall response to pembrolizumab (*p* = 0.042). It is noteworthy that we acquired and analyzed tumor biopsies immediately prior to the initiation of the treatment, preventing potential clonal selection of tumor cell subpopulations in respect to PD-L1 status. In summary, we performed a comprehensive characterization of HCC patients in response to pembrolizumab and identified potential immune-genomic correlates. We discovered that tumor cellular state as well as adjacent TME and circulating CD8+ T cells significantly contributed to the overall clinical response to pembrolizumab. HCC is a complex disease with diverse host-tumor interactions, often caused by chronic viral infection. Hence, we present empirical evidences to consider both tumor and host immune status to optimize immunotherapeutic treatment for HCC patients.

## Conclusions

Comprehensive genomic characterization of HCC identified a subset of patients with distinct molecular and immune profiles that respond to pembrolizumab, advocating the clinical feasibility of precision medical treatment in HCC.

## Supplementary Information

**Additional file 1:** The study protocol.

**Additional file 2:** The protocol revision history.

**Additional file 3: Table S1.** Clinicopathological information for pembrolizumab-treated HCC patients.

**Additional file 4: Table S2.** Clinicopathological features associated with response to pembrolizumab (*p* value by Fisher’s exact test). **Table S3.** Safety profiles. **Fig. S1.** Survival analysis. (A-B) PFS and OS of all patients (*n* = 60). (C-D) PFS and OS according to PR vs. SD/PD. (E-F) PFS and OS according to PD vs. PR/SD. **Fig. S2.** Prevalence of tumor-infiltrating lymphocytes (A) and PD-L1 expression level (B) based on *CTNNB1* somatic mutation. **Fig. S3.** Summary of nonsynonymous mutations in 47 HCC patients. **Fig. S4.** Correlation between the expression of neutrophil gene markers and NLR (A). Expression level of neutrophil gene markers according to NLR (B). **Fig. S5.** GSEA plots representing CHIANG_LIVER_CANCER_SUBCLASS_PROLIFERATION_UP pathway (A) and CHIANG_LIVER_CANCER_SUBCLASS_CTNNB1_DN pathway (B) were enriched in responders (PR). Patients with CTNNB1 mutation showed the higher expression levels of CTNNB1_UP geneset (C) and the lower expression levels of CTNNB1_DN geneset (D)

**Additional file 5: Table S4.** CTNNB1 mutations detected in pembrolizumab-treated patients.

## Data Availability

All types of raw sequencing data (WES, RNA-seq, and single-cell seq) have been deposited into European Nucleotide Archive (ENA) (primary accession number: PRJEB34724, secondary accession number: ERP117672) [53 https://www.ebi.ac.uk/ena/browser/view/PRJEB34724?show=reads
