# Supplementary material for: Hepatocellular carcinoma patients with high circulating cytotoxic T cells and intra-tumoral immune signature benefit from pembrolizumab: results from a single-arm phase 2 trial
Source: Genome Med. 2022 Jan 6;14:1. doi: 10.1186/s13073-021-00995-8 (PMC8734300; doi:10.1186/s13073-021-00995-8)
Supplement: Supplementary file 1 — Additional file 1: The study protocol. [file 13073_2021_995_MOESM1_ESM.pdf]

TITLE:

Phase II study of pembrolizumab in advanced hepatocellular carcinoma as second-line treatment after failure of sorafenib: integration of genomic analysis to identify predictive molecular subtypes

**Study Drug**	**pembrolizumab**
**Version Number**	**1.2**
**Version Date**	**21 November 2016**
**Study site**	**Samsung Medical Center**
**Principal Investigator (Sponsor-Investigator)**	**Professor Jeeyun Lee M.D, Ph.D.**

Revision History

Version
Version
Version 1.2 21 Nov 2016

PROTOCOL AGREEMENT

I have read the protocol specified below. In my formal capacity as Investigator, my duties include ensuring the safety of the study subjects enrolled under my supervision with complete and timely information, as outlined in the protocol. It is understood that all information pertaining to the study will be held strictly confidential and that this confidentiality requirement applies to all study staff at this site. Furthermore, on behalf of the study staff and myself, I agree to maintain the procedures required to carry out the study in accordance with accepted GCP principles and to abide by the terms of this protocol.

Protocol Title: *Phase II study of pembrolizumab in advanced hepatocellular carcinoma as second-line treatment after failure of sorafenib: integration of genomic analysis to identify predictive molecular subtypes*

____ ____________________

Investigator Signature *Date*

1.0 TRIAL SUMMARY

Abbreviated Title	*Phase II study of pembrolizumab in advanced hepatocellular carcinoma as second-line treatment after failure of sorafenib: integration of genomic analysis to identify predictive molecular subtypes*
Trial Phase	*II*
Clinical Indication	*Advanced hepatocellular carcinoma*
Trial Type	*Interventional*
Type of control	*One arm*
Route of administration	*Intravenous*
Trial Blinding	*Open-label*
Treatment Groups	*Pembrolizumab (MK-3475) 200 mg every 3 weeks (Q3W)*
Number of trial subjects	*Approximately up to 60 subjects will be enrolled.*
Estimated enrollment period	*12 months*
Estimated duration of trial	*Investigator estimates that the trial will require approximately*<br><br>*24 months from the time the first subject signs the informed consent until last subject's last visit.*<br>*Estimated date of first subject enrolled : Q4 2016*<br>*Estimated date of last subject completed: Q4 2017*
Duration of Participation	*Each subject will participate in the trial from the time the subject signs the Informed Consent Form (ICF) through the final contact. After a screening phase of up to 28 days, eligible subjects will receive treatment beginning on Day 1 of each 3week dosing cycle for pembrolizumab.*<br>*Treatment with pembrolizumab will continue until documented disease progression, unacceptable adverse event(s),intercurrent illness that prevents further administration of treatment, investigators' decision to withdraw the subject, subject withdraws consent, pregnancy of the subject, noncompliance with trial treatment or procedure requirements, subject receives 24 months of pembrolizumab, or administrative reasons requiring cessation of treatment. After the end of treatment, each subject will be followed for 30 days for adverse event monitoring (serious adverse events and events of clinical interest will be collected for 90 days after the end of treatment or 30 days after the end of treatment if the subject initiates new anticancer therapy, whichever is earlier).*<br>*Subjects who discontinue after 24months of therapy for reasons other tha n disease progression or intolerability or who discontinue after attaining a CR may be eligible for up to one year of retreatment after they have experienced radiographic disease progression.*<br>*Subjects who discontinue for reasons other than disease progression will have post-treatment follow-up for disease status until disease progression, initiating a non-study cancer treatment, withdrawing consent, or becoming lost to follow-up. All subjects will be followed by telephone for overall survival until death, withdrawal of consent, or the end of the study.*

Estimated average length of treatment per patient	*About 9~12 weeks*

TABLE OF CONTENTS

1.0 TRIAL SUMMARY 3
2.0 Title of the study 7
3.0 Participating Center 7
4.0 name of Principal Investigator, sub-investigators 7
4.1 Principal Investigator 7
4.2 Sub-Investigators 7
5.0 TRIAL DESIGN 7
5.1 Trial Design 7
5.2 Trial Diagram 8
6.0 OBJECTIVE(S) & HYPOTHESIS(ES) 9
6.1 Primary efficacy Objective(s) & Hypothesis(es) 9
6.2 Primary genomic Objective(s) & Hypothesis(es) 9
6.3 Exploratory Objective 9
7.0 BACKGROUND & RATIONALE 10
7.1 Background 10
7.2 Rationale 11
8.0 METHODOLOGY 16
8.1 Entry Criteria 16
8.2 Trial Treatments 20
8.3 Concomitant Medications/Vaccinations (allowed & prohibited) 22
8.4 Rescue Medications & Supportive Care 23
8.5 Diet/Activity/Other Considerations 27
8.6 Subject Withdrawal/Discontinuation Criteria 29
8.7 Subject Replacement Strategy 30
8.8 Clinical Criteria for Early Trial Termination 31

9.0 TRIAL FLOW CHART 32
9.1 Study Flow Chart 32
10.0 TRIAL PROCEDURES 35
10.1 Trial Procedures 35
10.2 Assessing and Recording Adverse Events..41
11.0 STATISTICAL ANALYSIS PLAN 50
11.1 Statistical Analysis Plan 50
11.2 Sample size..50
12.0 LABELING, PACKAGING, STORAGE AND RETURN OF CLINICAL SUPPLIES 51
12.1 Investigational Product..51
12.2 Packaging and Labeling Information 51
12.3 Clinical Supplies Disclosure 51
12.4 Storage and Handling Requirements..52
12.5 Returns and Reconciliation 52
13.0 ADMINISTRATIVE AND REGULATORY DETAILS..52
13.1 Confidentiality..52
13.2 Compliance with Financial Disclosure Requirements..53
13.3 Compliance with Law, Audit and Debarment 53
13.4 Compliance with Trial Registration and Results Posting Requirements 53
14.0 Monitoring 53
15.0 Data Handling 53
16.0 Sample Identification and retension specimen 53
17.0 References..54
18.0 APPENDICES 54
18.1 ECOG Performance Status..55
18.2 Common Terminology Criteria for Adverse Events V4.0 (CTCAE) 55

2.0 TITLE OF THE STUDY

Phase II study of pembrolizumab in advanced hepatocellular carcinoma as second-line treatment after failure of sorafenib: integration of genomic analysis to identify predictive molecular subtypes

3.0 PARTICIPATING CENTER

Division of Hematology-Oncology, Department of Medicine, Samsung Medical Center, Sungkyunkwan University School of Medicine, Seoul, Korea

4.0 NAME OF PRINCIPAL INVESTIGATOR, SUB-INVESTIGATORS

4.1 Principal Investigator

Professor Jeeyun Lee, M.D

4.2 Sub-Investigators

Ho Yeong Lim M.D

Seung Tae Kim, M.D.

Su Jin Lee, M.D

Yong Han Paik, M.D.

Wonsuk Kang, M.D.

5.0 TRIAL DESIGN

5.1 Trial Design

This is a single-arm, single-center, open-label trial of pembrolizumab (MK-3475) in subjects with advanced hepatocellular carcinoma as second-line treatment after failure of sorafenib.

Approximately 60 subjects will be enrollment to evaluate the efficacy and safety of pembrolizumab.

Enrollment will begin with all subjects without regard for PD-L1 expression status.

An evaluable specimen for PD-L1 status must be available and confirmed prior to enrollment.

All study subjects will be evaluated every 6 weeks (+/- 7 days) following the date of IP drug adminstration for the first six months and every 12 weeks (+/- 7 days) thereafter until progression

of disease is documented with radiologic imaging (computed tomography or magnetic resonance imaging).

The primary efficacy endpoint is ORR (objective response rate) per mRECIST.
If a subject has progression of disease by mRECIST, it is recommended that the subject be discontinued from the study treatment unless, in the Investigator's opinion, the subject is deriving benefit from treatment.
Clinically stable subjects may continue to receive trial therapy at the discretion of the Investigator. If a repeat scan confirms progression of disease and the subject remains clinically stable, the subject may continue treatment .

Adverse events will be monitored throughout the trial and graded in severity according to the guidelines outlined in the NCI Common Terminology Criteria for Adverse Events (CTCAE) version 4.0.

Except as noted above, treatment with pembrolizumab will continue until documented disease progression, unacceptable adverse event(s), intercurrent illness that prevents further administration of treatment, Investigator's decision to withdraw the subject, subject withdraws consent, pregnancy of the subject, noncompliance with trial treatment or procedure requirements, completion of 24 months of pembrolizumab, or administrative reasons requiring the cessation of treatment.

After the end of treatment, each subject will be followed for 30 days for adverse event monitoring (serious adverse events and events of clinical interest will be collected for 90 days after the end of treatment or 30 days after the end of treatment if the subject initiates new anticancer therapy, whichever is earlier).Subjects who discontinue treatment for reasons other than disease progression will have post-treatment follow-up for disease status until disease progression, initiating a non-study cancer treatment, withdrawing consent, or becoming lost to follow-up. All subjects will be followed by telephone contact for overall survival until death, withdrawal of consent or the end of the study, whichever comes first.

This study will be conducted in conformance with Good Clinical Practices.
Specific procedures to be performed during the trial, as well as their prescribed times and associated visit windows, are outlined in the Trial Flow Chart. Details of each procedure are provided in Trial Procedures.

5.2 Trial Scheme

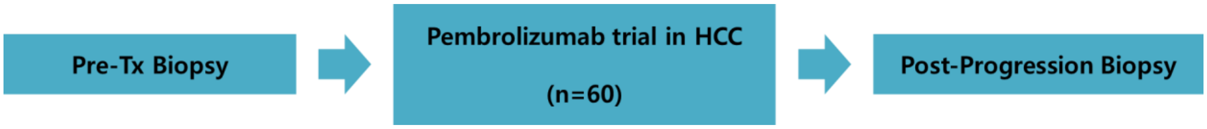

6.0 OBJECTIVE(S) & HYPOTHESIS(ES)

6.1 Primary efficacy Objective(s) & Hypothesis(es)

Objective: To evaluate RR per mRECIST in advanced hepatocellular carcinoma who have progressed on sorafenib, when treated with pembrolizumab

Hypothesis: Pembrolizumab increases RR per mRECIST in advanced hepatocellular carcinoma who have progressed on sorafenib

6.2 Primary genomic Objective(s) & Hypotheses

(1) **Primary objective:** Integrative genomic analysis to identify association of response (BOR, PFS and OS) and primary resistance to pembrolizumab with key genomic markers for immunotherapy (expression of PD-L1, T-cell inflamed and cytolytic immune signatures as measured by RNA-seq and/or Nanostring, MSI-h status, mutation load, and pathogen covariates, in particular Hepatitis viruses)

(2) **Hypothesis:**

1. Pembrolizumab will be effective in hepatocellular carcinoma
2. RNA-based cytolytic gene markers and immune signatures will associate with response to Pembrolizumab
3. Increased mutation load will associate with Pembrolizumab response
4. Pathogen infection will associate with Pembrolizumab response

6.3 Exploratory Objectives

(1) Integrated Genomic Analyses will be performed in an exploratory fashion to determine potential immune correlates of response to pembrolizumab at baseline and post-treatment (estimated 20% of post-treatment tumor samples will be available). The key components of this integrated analysis will be WES in the tumor (with matched normal) at baseline, and RNA-seq and Nanostring in tumors and blood pre and post treatment.

(2) NGS profiling will be performed at SMC and analyzed together by the BI team at MSD (Dr. Cristescu's group) and SMC (Genome Center and Molecular Pathology).

(3) As an exploratory analysis, we will isolate T cells using FACS sorting and sequence these cells. In addition, in order to identify the relationship between neoantigen drivers like infection and response to pembrolizumab, we will prospectively collect serum antigens from blood at baseline.

(4) Preplanned analysis: ctDNA analysis (pre, prior to cycle 3, and at progression) is planned in every patient responder vs non-responder will be analyzed.

7.0 BACKGROUND & RATIONALE

7.1 Background

Refer to the Investigator's Brochure (IB)/approved labeling for detailed background information on MK-3475.

7.1.1 Pharmaceutical and Therapeutic Background

The importance of intact immune surveillance in controlling outgrowth of neoplastic transformation has been known for decades. Accumulating evidence shows a correlation between tumor-infiltrating lymphocytes (TILs) in cancer tissue and favorable prognosis in various malignancies. In particular, the presence of CD8+ T-cells and the ratio of CD8+ effector T-cells / FoxP3+ regulatory T-cells seems to correlate with improved prognosis and long-term survival in many solid tumors.

The PD-1 receptor-ligand interaction is a major pathway hijacked by tumors to suppress immune control. The normal function of PD-1, expressed on the cell surface of activated T-cells under healthy conditions, is to down-modulate unwanted or excessive immune responses, including autoimmune reactions. PD-1 (encoded by the gene Pdcd1) is an Ig superfamily member related to CD28 and CTLA-4 which has been shown to negatively regulate antigen receptor signaling upon engagement of its ligands (PD-L1 and/or PD-L2). The structure of murine PD-1 has been resolved. PD-1 and family members are type I transmembrane glycoproteins containing an Ig Variable-type (V-type) domain responsible for ligand binding and a cytoplasmic tail which is responsible for the binding of signaling molecules. The cytoplasmic tail of PD-1 contains 2 tyrosine-based signaling motifs, an immunoreceptor tyrosine-based inhibition motif (ITIM) and an immunoreceptor tyrosine-based switch motif (ITSM). Following T-cell stimulation, PD-1 recruits the tyrosine phosphatases SHP-1 and SHP-2 to the ITSM motif within its cytoplasmic tail, leading to the dephosphorylation of effector molecules such as CD3ζ, PKCθ and ZAP70 which are involved in the CD3 T-cell signaling cascade. The mechanism by which PD-1 down modulates T-cell responses is similar to, but distinct from that of CTLA-4 as both molecules regulate an overlapping set of signaling proteins. PD-1 was shown to be expressed on activated lymphocytes including peripheral CD4+ and CD8+ T-cells, B-cells, T regs and Natural Killer cells. Expression has also been shown during thymic development on CD4-CD8- (double negative) T-cells as well as subsets of macrophages and dendritic cells. The ligands for PD-1 (PD-L1 and PD-L2) are constitutively expressed or can be induced in a variety of cell types, including non-hematopoietic tissues as well as in various tumors. Both ligands are type I transmembrane receptors containing both IgV- and IgC-like domains in the extracellular region and contain short cytoplasmic regions with no known signaling motifs. Binding of either PD-1 ligand to PD-1 inhibits T-cell activation triggered through the T-cell receptor. PD-L1 is expressed at low levels on various non-hematopoietic tissues, most notably on vascular endothelium, whereas PD-L2 protein is only detectably expressed on antigen-presenting cells found in lymphoid tissue or chronic inflammatory environments. PD-L2 is thought to control immune T-cell activation in lymphoid organs, whereas PD-L1 serves to dampen unwarranted T-cell function in peripheral tissues. Although healthy organs express little (if any) PD-L1, a variety of cancers were demonstrated to express abundant levels of this T-cell inhibitor. PD-1 has

been suggested to regulate tumor-specific T-cell expansion in subjects with melanoma (MEL). In gastric cancer PD-L1 and PD-L2 overexpression have recently been associated with EBV-positive tumors. This suggests that the PD-1/PD-L1 pathway plays a critical role in tumor immune evasion and should be considered as an attractive target for therapeutic intervention.

Pembrolizumab is a potent and highly selective humanized monoclonal antibody (mAb) of the IgG4/kappa isotype designed to directly block the interaction between PD-1 and its ligands, PD-L1 and PD-L2. Keytruda^{TM} (pembrolizumab) has recently been approved in the United Stated for the treatment of patients with unresectable or metastatic melanoma and disease progression following ipilumumab and, if BRAF V600 mutation positive, a BRAF inhibitor.

7.1.2 Preclinical and Clinical Trial Data

Refer to the Investigator's Brochure for Preclinical and Clinical data.

7.2 Rationale

7.2.1 Rationale for the Trial and Selected Subject Population

Primary tumors of the liver now represent the fifth most frequently diagnosed type of cancer worldwide, but the second most frequent cause of cancer death [1]. More than 75% of cases occur in the Asia-Pacific region, largely in association with chronic hepatitis B virus (HBV) infection [2]. Surgical resection leads to a 60–70% 5-year survival for patients with hepatocellular carcinoma (HCC) who present with solitary tumor and have excellent liver function. However, surgical resection is only an option for less than 20% of patients [3]. Disease that is diagnosed at an advanced stage or with progression after locoregional therapy has a dismal prognosis, owing to the underlying liver disease and lack of effective treatment options [4]. The only systemic treatment resulting in significant improvement in survival in patients with advanced HCC is sorafenib, a multi-kinase inhibitor targeting RAF kinase and receptor tyrosine kinases (RTKs), including platelet-derived growth factor receptor (PDGFR), vascular endothelial growth factor receptor (VEGFR), and c-KIT (a receptor specific for stem cell factor)[5,6]. Sorafenib simultaneously inhibits two key pathways that are reported to play an important role in the pathogenesis of HCC including regulation of tumor growth (via Raf-MEK-ERK) and angiogenesis (via VEGFR and PDGFR) [7]. Despite the encouraging achievement, sorafenib treatment was only 2-3months longer than placebo with a low partial response rate (3%) [5,6].

Starting 2014, SMC has launched a clinical sequencing program for the first time in Korea for oncology patients using targeted sequencing. The SMC team has extensive experience regarding clinical sequencing, including fresh biopsies, cfDNA, sequence reporting system etc. The NEXT-1 trial is a master protocol to route participants to different candidate drugs in trials based on clinical sequencing report. In this trial, we used a customized targeted enrichment panel consisting of cancer-related genes to interrogate single nucleotide variants (SNVs), insertions and deletions (Indels), copy number variations (CNVs) and a subset of gene fusions. Somatic mutations profiled by this targeted deep sequencing approach were classified into three tiers based on their clinical or biological significance, and guided oncologists in selection

of individualized treatments. In this master protocol, the response rate was assessed as the primary end point in patients who had molecularly-matched or standard therapy. From August 2014 through April 2015, 541 patients consented to participate at a single center in precision oncology clinic, and 419 cancer patients had sequencing data available to clinician for guidance to matched trials. The patient cohorts were gastric cancer (N = 127), colorectal cancer (N =123), pancreatic/biliary tract cancer (N=62) and rare cancers (N = 67). Out of 419 cancer patients, 260 (62.1%) patients either had no genomic alterations or no available matched trial, and 59 (22.7%) patients were successfully routed to genome-based matched clinical trial. In gastric cancer, the response rate of molecularly-matched group was significantly higher than that of the conventional treatment group (28.1% versus 5.9%, P<0.001). In particular, 16 rare cancer patients with matched therapy showed better response than cancer patients with the conventional salvage treatment (56.2% versus 12.5%, respectively; P=0.003). This was the first such master protocol reported in Asia. Through this trial, SMC has established a platform and a team where we can reliably perform upfront biopsy and sequencing.

In addition, SMC has previously worked with Pfizer to profile 286 HCC patients and has defined a predictive gene signature for tumor recurrence [8]. We identified 29 recurrently amplified and 22 recurrently deleted regions with a high level of copy number changes. These regions harbor established oncogenes and tumor suppressors, including CCND1 (cyclin D1), MET (hepatocyte growth factor receptor), CDKN2A (cyclin-dependent kinase inhibitor 2A) and CDKN2B (cyclin-dependent kinase inhibitor 2B), as well as many other genes not previously reported to be involved in liver carcinogenesis [9].

Recently [ASCO 2015], a phase I/II study of nivolumab has been reported. Patients enrolled in the study were required to have histologically confirmed advanced HCC with a Child-Pugh score of 7 or less and not be candidates for curative treatment options. Patients were allowed to have one or more prior lines of systemic therapy that failed, including sorafenib, the current standard of care in advanced HCC. In this dose-escalation study, patients received 0.1 to 10.0 mg/kg of nivolumab intravenously for up to 2 years. Dose escalation occurred in parallel cohorts based on whether they had hepatitis B (11 patients), hepatitis C (12 patients), or were noninfected (24 patients). Safety was the primary endpoint of the study. Response was measured using modified Response Evaluation Criteria in Solid Tumors (RECIST 1.1). Of 42 patients evaluable for response, CR was reported in two patients (5%) and partial response (PR) in six patients (14%) for an overall objective response rate of 19%. Responses were seen across all three cohorts (HBV, HCV, non-B non-C) of patients, and responses were durable. Preliminary 1-year overall survival (OS) was 62%. These responses were much higher than those seen with sorafenib (http://am.asco.org/nivolumab-hcc-and-pembrolizumab-tumors-mismatch-repair-deficiency). However, the molecular profile of the responders is currently unknown. Since not all HCC patients respond to anti-PD1 treatment, it is desirable to understand the molecular factors that affect response in order to design potential patient-tailored strategies of treatment with immune-modulating therapies such as anti-PD1.

A comprehensive molecular analysis of HBV-infected HCC was previously performed in the context of the ACRG collaboration (Asian cancer research group; a consortium of academic and pharma partners that included Merck and SMC) [10,11]. As part of that analysis, a subtype of HCC (S1) has been identified with particularly poor prognosis. A landmark of that subtype is the presence of immune markers consistent with T-cell infiltration of the tumor and cytolytic activity. Such a signature has been previously associated with response to pembrolizumab in multiple cancer types, including gastric cancer [12]. Given the dismal prognosis of the subjects in HCC group S1, we hypothesize that elements of the signature will also be enriched in sorafenib-refractory tumors and will indicate a potential for increased response to Pembrolizumab.

Hence, in this study, we propose to identify for the first time DNA- and RNA-based molecular as well as etiology subtypes of sorafenib-refractory HCC who may respond to pembrolizumab, by building upon the previous successful ACRG collaborative work in Gastric cancer [13] between MERCK MSD and SMC team.

7.2.2 Rationale for Dose Selection/Regimen/Modification

An open-label Phase I trial (Protocol 001) is being conducted to evaluate the safety and clinical activity of single agent MK-3475. The dose escalation portion of this trial evaluated three dose levels, 1 mg/kg, 3 mg/kg, and 10 mg/kg, administered every 2 weeks (Q2W) in subjects with advanced solid tumors. All three dose levels were well tolerated and no dose-limiting toxicities were observed. This first in human study of MK-3475 showed evidence of target engagement and objective evidence of tumor size reduction at all dose levels (1 mg/kg, 3 mg/kg and 10 mg/kg Q2W). No MTD has been identified to date. 10.0 mg/kg Q2W, the highest dose tested in PN001, will be the dose and schedule utilized in Cohorts A, B, C and D of this protocol to test for initial tumor activity. Recent data from other clinical studies within the MK-3475 program has shown that a lower dose of MK-3475 and a less frequent schedule may be sufficient for target engagement and clinical activity.

PK data analysis of MK-3475 administered Q2W and Q3W showed slow systemic clearance, limited volume of distribution, and a long half-life (refer to IB). Pharmacodynamic data (IL-2 release assay) suggested that peripheral target engagement is durable (>21 days). This early PK and pharmacodynamic data provides scientific rationale for testing a Q2W and Q3W dosing schedule.

A population pharmacokinetic analysis has been performed using serum concentration time data from 476 patients. Within the resulting population PK model, clearance and volume parameters of MK-3475 were found to be dependent on body weight. The relationship between clearance and body weight, with an allometric exponent of 0.59, is within the range observed for other antibodies and would support both body weight normalized dosing or a fixed dose across all body weights. MK-3475 has been found to have a wide therapeutic range based on the melanoma indication. The differences in exposure for a 200 mg fixed dose regimen relative

to a 2 mg/kg Q3W body weight based regimen are anticipated to remain well within the established exposure margins of 0.5 – 5.0 for MK-3475 in the melanoma indication. The exposure margins are based on the notion of similar efficacy and safety in melanoma at 10 mg/kg Q3W vs. the proposed dose regimen of 2 mg/kg Q3W (i.e. 5-fold higher dose and exposure). The population PK evaluation revealed that there was no significant impact of tumor burden on exposure. In addition, exposure was similar between the NSCLC and melanoma indications. Therefore, there are no anticipated changes in exposure between different indication settings.

The rationale for further exploration of 2 mg/kg and comparable doses of pembrolizumab in solid tumors is based on: 1) similar efficacy and safety of pembrolizumab when dosed at either 2 mg/kg or 10 mg/kg Q3W in melanoma patients, 2) the flat exposure-response relationships of pembrolizumab for both efficacy and safety in the dose ranges of 2 mg/kg Q3W to 10 mg/kg Q3W, 3) the lack of effect of tumor burden or indication on distribution behavior of pembrolizumab (as assessed by the population PK model) and 4) the assumption that the dynamics of pembrolizumab target engagement will not vary meaningfully with tumor type.

The choice of the 200 mg Q3W as an appropriate dose for the switch to fixed dosing is based on simulations performed using the population PK model of pembrolizumab showing that the fixed dose of 200 mg every 3 weeks will provide exposures that 1) are optimally consistent with those obtained with the 2 mg/kg dose every 3 weeks, 2) will maintain individual patient exposures in the exposure range established in melanoma as associated with maximal efficacy response and 3) will maintain individual patients exposure in the exposure range established in melanoma that are well tolerated and safe.

A fixed dose regimen will simplify the dosing regimen to be more convenient for physicians and to reduce potential for dosing errors. A fixed dosing scheme will also reduce complexity in the logistical chain at treatment facilities and reduce wastage.

7.2.3 Rationale for Endpoints

7.2.3.1 Efficacy Endpoints

mRECIST will be adapted to account for the unique tumor response profile seen with immunotherapies such as pembrolizumab. Immunotherapeutic agents such as pembrolizumab may produce antitumor effects by potentiating endogenous cancer-specific immune responses which may be functionally anergic. The response patterns seen with such an approach may extend beyond the typical time course of responses seen with cytotoxic agents, and can manifest a clinical response after an initial increase in tumor burden or even the appearance of new lesions.

When feasible, subjects within the pembrolizumab arm should not be discontinued until progression is confirmed. This allowance to continue treatment despite initial radiologic progression takes into account the observation that some subjects can have a transient tumor flare in the first few months after the start of immunotherapy, but with subsequent disease response.

7.2.3.2 Biomarker Research

The key goal of this effort is to determine genomic correlates of response to pembrolizumab in HCC. Nivolumab activity has been reported in HCC and genomic characterization of HCC has been a part of large scale cancer characterization such as TCGA and ACRG. Moreover, important *independent and universal* key genomic markers of response have been identified in multiple tumor types, in particular RNA based (T-cell inflamed signature) and DNA based (mutational load). However, to the best of our knowledge these interactions and associations have not been explored to date in the case of HCC. Although our effort will be focused on patients refractory to SOC, it is expected that identification of such biomarkers will importantly impact clinical strategies also for earlier stages of disease.

To characterize the tumors in this protocol we will perform tumor NGS sequencing (RNA sequencing for estimating the T-cell inflamed signature and other immunogical covariates, WES and/or TES cancer panel for estimating the mutation load and other somatic covariates), as well as IHC for key markers like PDL1, FOXP3, CD4, CD8 and MLH1.

We expect that 20% of the patients will also have post-treatment tumors available for which similar NGS characterization will be performed (WES and RNA-seq), with the goal of identifying potential tumor-specific mechanisms of acquired resistance to pembrolizumab in patients with progressive disease after initial response.

As an exploratory analysis, we will isolate T cells using FACS sorting and sequence these cells. In addition, in order to identify the relationship between neoantigen drivers like infection and response to pembrolizumab, we will prospectively collect serum antigens from blood at baseline.

Immune checkpoint molecules are dysregulated in HCC, most notably PD-1, PD-L1, CTLA-4, TIM-3, KIR, and LAG3. Although all these molecules can potentially be leveraged for therapeutic benefit, the PD-1 and PD-L1 axis is the most topical. PD-L1 is heavily expressed in HCC and surrounding PACs (LSECs, KCs, and tumor-associated monocytes). Retrospective studies have indicated that PD-L1 expression, as assessed by flow cytometry, Western blot analysis, and immunohistochemical staining, ranges from 45% to 100% in HCC samples. In a cohort of 240 patients with surgically resected HCC, tumoral PD-L1 expression was associated with aggressive clinicopathologic features and statistically significantly shorter disease-free survival [14]. Certain HCC etiologic factors enrich for PD-L1 expression and it will be interesting to formally assess how expression changes across HCC subtypes [15].

Arrays of cytokine profiles (increased IL-4, 5, 8, and IL-10 secretion and relative suppression of IL-1, tumor necrosis factor, and IFN-r) in the HCC microenvironment results in blunting of the normally protective T-helper 1 immune response needed to effectively combat malignancy [16]. This cytokine signature is associated with a poor prognosis and aggressive disease characteristics. High levels of circulating TGF-b also portend inferior survival in patients with HCC. Although the tumor-promoting effects of TGF-b include enhanced neovascularization,

the promotion of metastasis, and induction of fibrosis, there is clear evidence that this molecule is immunosuppressive. TGF-b induces Treg polarization and differentiation, and is a key negative regulator of CD81T cells, promoting T-cell exhaustion.

For HCC, pembrolizumab has not been extensively tested in terms of efficacy, especially in correlation with genomic profiling. Thus, herein we aim to identify for the first time DNA- and RNA-based molecular as well as etiology subtypes of Asian-specific HCC who may respond to pembrolizumab.

8.0 METHODOLOGY

8.1 Entry Criteria

8.1.1 Diagnosis/Condition for Entry into the Trial

8.1.2 Subject Inclusion Criteria

In order to be eligible for participation in this trial, the subject must:

1. Be willing and able to provide written informed consent/assent for the trial. The subject may also provide consent for Biomedical Research. However, the subject may participate in the main trial without participating in Biomedical Research.

2. Be ≥ 20 years of age on day of signing informed consent (or acceptable age according to local regulations, whichever is older).

3. Have histologically or cytologically confirmed diagnosis of HCC (fibrolamellar and mixed hepatocellular/cholangiocarcinoma subtypes are not eligible) based on pathology report.

4. Have Barcelona Clinic Liver Cancer (BCLC) Stage C disease, or BCLC Stage B disease not amenable to locoregional therapy or refractory to locoregional therapy, and not amenable to a curative treatment approach.

5. Have a Child-Pugh class A liver score

6. Has experienced documented objective radiographic or clinical disease progression during first-line sorafenib therapy.

7. Have measurable disease based on mRECIST as determined by investigator. Tumor lesions situated in a previously irradiated area are considered measurable if progression has been demonstrated in such lesions.
    a. Note: The exact same image acquisition and processing parameters should be used throughout the study.

8. Be willing to provide fresh tissue for biomarker analysis, and, based on the adequacy of the tissue sample quality for assessment of biomarker status. Repeat samples may be required if adequate tissue is not provided. Newly obtained endoscopic biopsy specimens are preferred to archived samples and formalin-fixed, paraffin-embedded (FFPE) block specimens are preferred to slides.

   a. *Newly-obtained is defined as a specimen obtained up to 6 weeks (42 days) prior to initiation of treatment on Day 1. Subjects for whom newly-obtained samples cannot be provided (e.g. inaccessible or subject safety concern) may submit an archived specimen.*

   b. *Collection of an archived tissue sample will also be requested (where available) to support evaluation of the clinical utility of biomarker assessment in newly obtained vs. archived tissue samples; however, a subject will not be precluded from participating in the study if an archived tissue sample is not available for collection or is otherwise insufficient for analysis.*

9. Have a performance status of 0 or 1 on the ECOG Performance Scale.

10. Subjects are eligible to enroll if they have non-viral-HCC, or if they have HBV-HCC, or HCV-HCC defined as follows:

   i) HBV-HCC: Resolved HBV infection (as evidenced by detectable HBV surface antibody, detectable HBV core antibody, undetectable HBV DNA, and undetectable HBV surface antigen) or Chronic HBV infection (as evidenced by detectable HBV surface antigen or HBV DNA). Subjects with chronic HBV infection must have HBV DNA < 100 IU/mL and must be on antiviral therapy.

   ii) HCV-HCC: Active or resolved HCV infection as evidenced by detectable HCV RNA or antibody.

11. Demonstrate adequate organ function as defined in Table 1, all screening labs should be performed within 10 days of treatment initiation.

Table 1 Adequate Organ Function Laboratory Values

System	Laboratory Value
**Hematological**	
Absolute neutrophil count (ANC)	≥1,200 /mcL
Platelets	≥60,000 / mcL
Hemoglobin	≥8 g/dL or ≥5.6 mmol/L without transfusion or EPO dependency (within 7 days of assessment)
**Renal**	
Serum creatinine **<u>OR</u>**<br>Measured or calculated[a] creatinine clearance	≤1.5 X upper limit of normal (ULN) **<u>OR</u>**<br><br>≥60 mL/min for subject with creatinine levels > 1.5 X institutional ULN

(GFR can also be used in place of creatinine or CrCl)	
**Hepatic**	
Serum total bilirubin	≤ 2 X ULN **OR**
	Direct bilirubin ≤ ULN for subjects with total bilirubin levels > 2 ULN
AST (SGOT) and ALT (SGPT)	≤ 5 X ULN
Albumin	≥2.5 mg/dL
**Coagulation**	
International Normalized Ratio (INR) or Prothrombin Time (PT)	≤1.5 X ULN unless subject is receiving anticoagulant therapy as long as PT or PTT is within therapeutic range of intended use of anticoagulants
Activated Partial Thromboplastin Time (aPTT)	≤1.5 X ULN unless subject is receiving anticoagulant therapy as long as PT or PTT is within therapeutic range of intended use of anticoagulants
[a]Creatinine clearance should be calculated per institutional standard.	

12. Female subject of childbearing potential should have a negative urine or serum pregnancy within 72 hours prior to receiving the first dose of study medication. If the urine test is positive or cannot be confirmed as negative, a serum pregnancy test will be required.

13. Female subjects of childbearing potential (Section 5.7.2) must be willing to use an adequate method of contraception as outlined in Section 5.7.2 – Contraception, for the course of the study through 120 days after the last dose of study medication.

Note: Abstinence is acceptable if this is the usual lifestyle and preferred contraception for the subject.

14. Male subjects of childbearing potential (Section 5.7.1) must agree to use an adequate method of contraception as outlined in Section 5.7.1- Contraception, starting with the first dose of study therapy through 120 days after the last dose of study therapy.

Note: Abstinence is acceptable if this is the usual lifestyle and preferred contraception for the subject.

8.1.3 Subject Exclusion Criteria

The subject must be excluded from participating in the trial if the subject:

1. Is currently participating and receiving study therapy or has participated in a study of an investigational agent and received study therapy or used an investigational device within 4 weeks of the first dose of treatment.

2. Has received sorafenib within 14 days of first dose of study medication.

3. Has had esophageal or gastric variceal bleeding withint the last 6 months. All subjects will be screened for esophageal varices, unless such screening has been performed in the past 12 months before first dose of treatment. If varices are present, they should be treated according to institutional standards before starting study treatment.

4. Had a solid organ transplant.

5. Has active autoimmune disease that has required systemic treatment in past 2 years (i.e., with use of disease-modifying agents, corticosteroids, or immunosuppressive drugs). Replacement therapy (e.g., thyroxine, insulin, or physiologic corticosteroid replacement therapy for adrenal or pituitary insufficiency, etc.) is not considered a form of systemic treatment.

6. Has a diagnosis of immunodeficiency or is receiving systemic steroid therapy or any other form of immunosuppressive therapy within 7 days prior to the first dose of trial treatment.

7. Has received locoregional therapy to liver (transcatheter chemoembolization [TACE], transcatheter embolization [TAE], radiation, radioembolization, or ablation) or major surgery to liver or other site within 6 weeks prior to the first dose of study drug. Minor surgery (e.g., simple excision, tooth extraction) must have occurred at least 7 days prior to the first dose of study treatment (Cycle 1, Day 1). Subjects must have recovered adequately (i.e., Grade ≤1 or baseline) from the toxicity and/or complications from any intervention prior to starting therapy.

8. Has a known additional malignancy that is progressing or requires active treatment. Exceptions include basal cell carcinoma of the skin or squamous cell carcinoma of the skin that has undergone potentially curative therapy or in situ cervical cancer.

9. Has known active central nervous system (CNS) metastases and/or carcinomatous meningitis. Subjects with previously treated brain metastases may participate provided they are stable (without evidence of progression by imaging for at least four weeks prior to the first dose of trial treatment and any neurologic symptoms have returned to baseline), have no evidence of new or enlarging brain metastases, and are not using steroids for at least 7 days prior to trial treatment. This exception does not include carcinomatous meningitis which is excluded regardless of clinical stability.

10. Has active autoimmune disease that has required systemic treatment in the past 2 years (i.e. with use of disease modifying agents, corticosteroids or immunosuppressive drugs). Replacement therapy (eg., thyroxine, insulin, or physiologic corticosteroid replacement therapy for adrenal or pituitary insufficiency, etc.) is not considered a form of systemic treatment.

11. Has a known history of, or any evidence of, interstitial lung disease or active noninfectious pneumonitis.

12. Has an active infection requiring systemic therapy.

13. Has a history or current evidence of any condition, therapy, or laboratory abnormality that might confound the results of the trial, interfere with the subject’s participation for the full duration of the trial, or is not in the best interest of the subject to participate, in the opinion of the treating investigator.

14. Has known psychiatric or substance abuse disorders that would interfere with cooperation with the requirements of the trial.

15. Is pregnant or breastfeeding, or expecting to conceive or father children within the projected duration of the trial, starting with the pre-screening or screening visit through 120 days after the last dose of trial treatment.

16. Has received prior therapy with an anti-PD-1, anti-PD-L1, or anti-PD-L2 agent.

17. Has a known history of Human Immunodeficiency Virus (HIV) (HIV 1/2 antibodies).

18. Has untreated active Hepatitis B (e.g., HBsAg reactive) or Hepatitis C (e.g., HCV RNA [qualitative] is detected).

Note: To qualify for enrollment, antiviral therapy for HBV must be given for at least 3 months, and HBV viral load must be less than 100 IU/mL prior to first dose of study drug. Those on active HBV therapy with viral loads under 100 IU/ml should stay on the same therapy throughout study treatment. Those subjects who are anti-HBc (+) and negative for HBsAg and negative for anti-HBs, and have an HBV viral load under 100 IU/mL do not require HBV anti-viral prophylaxis, but need close monitoring.

19. Has received a live vaccine within 30 days of planned start of study therapy.

Note: Seasonal influenza vaccines for injection are generally inactivated flu vaccines and are allowed; however intranasal influenza vaccines (e.g., Flu-Mist®) are live attenuated vaccines, and are not allowed.

8.2 Trial Treatments

The treatment to be used in this trial is outlined below in Table 2

Table 2 Trial Treatment

Drug	Dose/Potency	Dose Frequency	Route of Administration	Regimen/Treatment Period	Use
Pembrolizumab	200 mg	Q3W	IV infusion	Day 1 of each 3 week cycle	Experimental

8.2.1 Dose Selection/Modification

8.2.1.1 Dose Selection

The rationale for selection of doses to be used in this trial is provided in Background and Rationale.

8.2.1.2 Dose Modification (Escalation/Titration/Other)

Adverse events (both non-serious and serious) associated with pembrolizumab exposure may represent an immunologic etiology. These adverse events may occur shortly after the first dose or several months after the last dose of treatment. Pembrolizumab must be withheld for drug-related toxicities and severe or life-threatening AEs as per Table 3 below. See Section 8.4 for supportive care guidelines, including use of corticosteroids.

Table 3

Dose Modification Guidelines for Drug-Related Adverse Events

Toxicity	Hold Treatment For Grade	Timing for Restarting Treatment	Treatment Discontinuation
Diarrhea/Colitis	2-3	Toxicity resolves to Grade 0-1	Toxicity does not resolve within 12 weeks of last dose or inability to reduce corticosteroid to 10 mg or less of prednisone or equivalent per day within 12 weeks
	4	Permanently discontinue	Permanently discontinue
AST, ALT, or Increased Bilirubin	2	Toxicity resolves to Grade 0-1	Toxicity does not resolve within 12 weeks of last dose
	3-4	Permanently discontinue (see exception below)[a]	Permanently discontinue
Type 1 diabetes mellitus (if new onset) or Hyperglycemia	T1DM or 3-4	Hold pembrolizumab for new onset Type 1 diabetes mellitus or Grade 3-4 hyperglycemia associated with evidence of beta cell failure	Resume pembrolizumab when patients are clinically and metabolically stable
Hypophysitis	2-4	Toxicity resolves to Grade 0-1. Therapy with pembrolizumab can be continued while endocrine replacement therapy is instituted	Toxicity does not resolve within 12 weeks of last dose or inability to reduce corticosteroid to 10 mg or less of prednisone or equivalent per day within 12 weeks
Hyperthyroidism	3	Toxicity resolves to Grade 0-1	Toxicity does not resolve within 12 weeks of last dose or inability to reduce corticosteroid to 10 mg or less of prednisone or equivalent per day within 12 weeks
	4	Permanently discontinue	Permanently discontinue
Hypothyroidism		Therapy with pembrolizumab can be continued while thyroid replacement therapy is instituted	Therapy with pembrolizumab can be continued while thyroid replacement therapy is instituted
Infusion Reaction	2[b]	Toxicity resolves to Grade 0-1	Permanently discontinue if toxicity develops despite adequate premedication
	3-4	Permanently discontinue	Permanently discontinue
Pneumonitis	2	Toxicity resolves to Grade 0-1	Toxicity does not resolve within 12 weeks of last dose or inability to reduce corticosteroid to 10 mg or less of prednisone or equivalent per day within 12 weeks
	3-4	Permanently discontinue	Permanently discontinue
Renal Failure or Nephritis	2	Toxicity resolves to Grade 0-1	Toxicity does not resolve within 12 weeks of last dose or inability to reduce corticosteroid to 10 mg or less of prednisone or equivalent per day within 12 weeks
	3-4	Permanently discontinue	Permanently discontinue
All Other Drug-Related Toxicity[c]	3 or Severe	Toxicity resolves to Grade 0-1	Toxicity does not resolve within 12 weeks of last dose or inability to reduce corticosteroid to 10 mg or less of prednisone or equivalent per day within 12 weeks
	4	Permanently discontinue	Permanently discontinue

Note: Permanently discontinue for any severe or Grade 3 drug-related AE that recurs or any life-threatening event.

[a] For patients with liver metastasis who begin treatment with Grade 2 AST or ALT, if AST or ALT increases by greater than or equal to 50% relative to baseline and lasts for at least 1 week then patients should be discontinued.

[b] If symptoms resolve within one hour of stopping drug infusion, the infusion may be restarted at 50% of the original infusion rate (e.g., from 100 mL/hr to 50 mL/hr). Otherwise dosing will be held until symptoms resolve and the subject should be premedicated for the next scheduled dose; Refer to

Toxicity	Hold Treatment For Grade	Timing for Restarting Treatment	Treatment Discontinuation
Table – Infusion Treatment Guidelines for further management details.<br>[c] Patients with intolerable or persistent Grade 2 drug-related AE may hold study medication at physician discretion. Permanently discontinue study drug for persistent Grade 2 adverse reactions for which treatment with study drug has been held, that do not recover to Grade 0-1 within 12 weeks of the last dose.			

Dosing interruptions are permitted in the case of medical / surgical events or logistical reasons not related to study therapy (e.g., elective surgery, unrelated medical events, patient vacation, and/or holidays). Subjects should be placed back on study therapy within 3 weeks of the scheduled interruption. The reason for interruption should be documented in the patient's study record.

8.2.2 Timing of Dose Administration

Trial treatment should be administered on Day 1 of each cycle after all procedures/assessments have been completed as detailed on the Trial Flow Chart (Section 6.0). Trial treatment may be administered up to 3 days before or after the scheduled Day 1 of each cycle due to administrative reasons.

All trial treatments will be administered on an outpatient basis.

Pembrolizumab 200 mg will be administered as a 30 minute IV infusion every 3 weeks. Sites should make every effort to target infusion timing to be as close to 30 minutes as possible. However, given the variability of infusion pumps from site to site, a window of -5 minutes and +10 minutes is permitted (i.e., infusion time is 30 minutes: -5 min/+10 min).

8.2.3 Trial Blinding/Masking

This is an open-label trial; therefore, the investigator and subject will know the treatment administered.

8.3 Concomitant Medications/Vaccinations (allowed & prohibited)

Medications or vaccinations specifically prohibited in the exclusion criteria are not allowed during the ongoing trial. If there is a clinical indication for one of these or other medications or vaccinations specifically prohibited during the trial, discontinuation from trial therapy or vaccination may be required. The investigator should discuss any questions regarding this with the Merck Clinical team. The final decision on any supportive therapy or vaccination rests with the investigator and/or the subject's primary physician.

8.3.1 Acceptable Concomitant Medications

All treatments that the investigator considers necessary for a subject’s welfare may be administered at the discretion of the investigator in keeping with the community standards of medical care. All concomitant medication will be recorded on the case report form (CRF) including all prescription, over-the-counter (OTC), herbal supplements, and IV medications and fluids. If changes occur during the trial period, documentation of drug dosage, frequency, route, and date may also be included on the CRF.

All concomitant medications received within 28 days before the first dose of trial treatment and 30 days after the last dose of trial treatment should be recorded. Concomitant medications administered after 30 days after the last dose of trial treatment should be recorded for SAEs and ECIs as defined in Section 7.2.

8.3.2 Prohibited Concomitant Medications

Subjects are prohibited from receiving the following therapies during the Screening and Treatment Phase (including retreatment for post-complete response relapse) of this trial:

- Antineoplastic systemic chemotherapy or biological therapy
- Immunotherapy not specified in this protocol
- Chemotherapy not specified in this protocol
- Investigational agents other than pembrolizumab
- Radiation therapy
    - Note: Radiation therapy to a symptomatic solitary lesion or to the brain may be allowed at the investigator's discretion.
- Live vaccines within 30 days prior to the first dose of trial treatment and while participating in the trial. Examples of live vaccines include, but are not limited to, the following: measles, mumps, rubella, varicella/zoster, yellow fever, rabies, BCG, and typhoid vaccine.

- Systemic glucocorticoids for any purpose other than to modulate symptoms from an event of clinical interest of suspected immunologic etiology. The use of physiologic doses of corticosteroids may be approved after consultation with the Merk.

Subjects who, in the assessment by the investigator, require the use of any of the aforementioned treatments for clinical management should be removed from the trial. Subjects may receive other medications that the investigator deems to be medically necessary.

The Exclusion Criteria describes other medications which are prohibited in this trial.

There are no prohibited therapies during the Post-Treatment Follow-up Phase.

8.4 Rescue Medications & Supportive Care

8.4.1 Supportive Care Guidelines

Subjects should receive appropriate supportive care measures as deemed necessary by the treating investigator. Suggested supportive care measures for the management of adverse events with potential immunologic etiology are outlined below. Where appropriate, these guidelines include the use of oral or intravenous treatment with corticosteroids as well as additional anti-inflammatory agents if symptoms do not improve with administration of corticosteroids. Note that several courses of steroid tapering may be necessary as symptoms may worsen when the steroid dose is decreased. For each disorder, attempts should be made to

rule out other causes such as metastatic disease or bacterial or viral infection, which might require additional supportive care. The treatment guidelines are intended to be applied when the investigator determines the events to be related to pembrolizumab.

Note: if after the evaluation the event is determined not to be related, the investigator does not need to follow the treatment guidance (as outlined). Refer to Section 5.2.1 for dose modification.

It may be necessary to perform conditional procedures such as bronchoscopy, endoscopy, or skin photography as part of evaluation of the event.

- **Pneumonitis**:
    - For **Grade 2 events**, treat with systemic corticosteroids. When symptoms improve to Grade 1 or less, steroid taper should be started and continued over no less than 4 weeks.
    - For **Grade 3-4 events**, immediately treat with intravenous steroids. Administer additional anti-inflammatory measures, as needed.
    - Add prophylactic antibiotics for opportunistic infections in the case of prolonged steroid administration.

- **Diarrhea/Colitis**:
  Subjects should be carefully monitored for signs and symptoms of enterocolitis (such as diarrhea, abdominal pain, blood or mucus in stool, with or without fever) and of bowel perforation (such as peritoneal signs and ileus).

    - All subjects who experience diarrhea/colitis should be advised to drink liberal quantities of clear fluids. If sufficient oral fluid intake is not feasible, fluid and electrolytes should be substituted via IV infusion. For Grade 2 or higher diarrhea, consider GI consultation and endoscopy to confirm or rule out colitis.
    - For **Grade 2 diarrhea/colitis**, administer oral corticosteroids.
    - For **Grade 3 or 4 diarrhea/colitis**, treat with intravenous steroids followed by high dose oral steroids.
    - When symptoms improve to Grade 1 or less, steroid taper should be started and continued over no less than 4 weeks.

- **Type 1 diabetes mellitus (if new onset, including diabetic ketoacidosis [DKA]) or ≥ Grade 3 Hyperglycemia, if associated with ketosis (ketonuria) or metabolic acidosis (DKA)**

    - For **T1DM** or **Grade 3-4** Hyperglycemia
        - Insulin replacement therapy is recommended for Type I diabetes mellitus and for Grade 3-4 hyperglycemia associated with metabolic acidosis or ketonuria.
        - Evaluate patients with serum glucose and a metabolic panel, urine ketones, glycosylated hemoglobin, and C-peptide.

- **Hypophysitis**:

    - For **Grade 2** events, treat with corticosteroids. When symptoms improve to Grade 1 or less, steroid taper should be started and continued over no less than 4 weeks. Replacement of appropriate hormones may be required as the steroid dose is tapered.
    - For **Grade 3-4** events, treat with an initial dose of IV corticosteroids followed by oral corticosteroids. When symptoms improve to Grade 1 or less, steroid taper should be started and continued over no less than 4 weeks. Replacement of appropriate hormones may be required as the steroid dose is tapered.

- **Hyperthyroidism or Hypothyroidism**:
  Thyroid disorders can occur at any time during treatment. Monitor patients for changes in thyroid function (at the start of treatment, periodically during treatment, and as indicated based on clinical evaluation) and for clinical signs and symptoms of thyroid disorders.

    - **Grade 2** hyperthyroidism events (and **Grade 2-4** hypothyroidism):
        - In hyperthyroidism, non-selective beta-blockers (e.g. propranolol) are suggested as initial therapy.
        - In hypothyroidism, thyroid hormone replacement therapy, with levothyroxine or liothyroinine, is indicated per standard of care.
    - **Grade 3-4** hyperthyroidism
        - Treat with an initial dose of IV corticosteroid followed by oral corticosteroids. When symptoms improve to Grade 1 or less, steroid taper should be started and continued over no less than 4 weeks. Replacement of appropriate hormones may be required as the steroid dose is tapered.

- **Hepatic**:
    - For **Grade 2** events, monitor liver function tests more frequently until returned to baseline values (consider weekly).
        - Treat with IV or oral corticosteroids
    - For **Grade 3-4** events, treat with intravenous corticosteroids for 24 to 48 hours.
    - When symptoms improve to Grade 1 or less, a steroid taper should be started and continued over no less than 4 weeks.

- **Renal Failure or Nephritis**:
    - For **Grade 2** events, treat with corticosteroids.
    - For **Grade 3-4** events, treat with systemic corticosteroids.
    - When symptoms improve to Grade 1 or less, steroid taper should be started and continued over no less than 4 weeks.

- **Management of Infusion Reactions**: Signs and symptoms usually develop during or shortly after drug infusion and generally resolve completely within 24 hours of completion of infusion.

Table 4 below shows treatment guidelines for subjects who experience an infusion reaction associated with administration of pembrolizumab (MK-3475).

Table 4 Infusion Reaction Treatment Guidelines

NCI CTCAE Grade	Treatment	Premedication at subsequent dosing
Grade 1<br>Mild reaction; infusion interruption not indicated; intervention not indicated	Increase monitoring of vital signs as medically indicated until the subject is deemed medically stable in the opinion of the investigator.	None
Grade 2<br>Requires infusion interruption but responds promptly to symptomatic treatment (e.g., antihistamines, NSAIDS, narcotics, IV fluids); prophylactic medications indicated for < =24 hrs	**Stop Infusion and monitor symptoms.**<br>Additional appropriate medical therapy may include but is not limited to:<br>IV fluids<br>Antihistamines<br>NSAIDS<br>Acetaminophen<br>Narcotics<br>Increase monitoring of vital signs as medically indicated until the subject is deemed medically stable in the opinion of the investigator.<br>If symptoms resolve within one hour of stopping drug infusion, the infusion may be restarted at 50% of the original infusion rate (e.g., from 100 mL/hr to 50 mL/hr). Otherwise dosing will be held until symptoms resolve and the subject should be premedicated for the next scheduled dose.<br>**Subjects who develop Grade 2 toxicity despite adequate premedication should be permanently discontinued from further trial treatment administration.**	Subject may be premedicated 1.5h (± 30 minutes) prior to infusion of pembrolizumab (MK-3475) with:<br><br>Diphenhydramine 50 mg po (or equivalent dose of antihistamine).<br><br>Acetaminophen 500-1000 mg po (or equivalent dose of antipyretic).
Grades 3 or 4<br><br>Grade 3:<br>Prolonged (i.e., not rapidly responsive to symptomatic medication and/or brief interruption of infusion); recurrence of symptoms following initial improvement; hospitalization indicated for other clinical sequelae (e.g., renal impairment, pulmonary infiltrates)<br><br>Grade 4:<br>Life-threatening; pressor or ventilatory support indicated	**Stop Infusion.**<br>Additional appropriate medical therapy may include but is not limited to:<br>IV fluids<br>Antihistamines<br>NSAIDS<br>Acetaminophen<br>Narcotics<br>Oxygen<br>Pressors<br>Corticosteroids<br>Epinephrine<br><br>Increase monitoring of vital signs as medically indicated until the subject is deemed medically stable in the opinion of the investigator.<br>Hospitalization may be indicated.<br>**Subject is permanently discontinued from further trial treatment administration.**	No subsequent dosing

NCI CTCAE Grade	Treatment	Premedication at subsequent dosing
Appropriate resuscitation equipment should be available in the room and a physician readily available during the period of drug administration.		

8.5 Diet/Activity/Other Considerations

8.5.1 Diet

Subjects should maintain a normal diet unless modifications are required to manage an AE such as diarrhea, nausea or vomiting.

8.5.2 Contraception

Pembrolizumab may have adverse effects on a fetus in utero. Furthermore, it is not known if pembrolizumab has transient adverse effects on the composition of sperm.

For this trial, male subjects will be considered to be of non-reproductive potential if they have azoospermia (whether due to having had a vasectomy or due to an underlying medical condition).

Female subjects will be considered of non-reproductive potential if they are either:

(1) postmenopausal (defined as at least 12 months with no menses without an alternative medical cause; in women < 45 years of age a high follicle stimulating hormone (FSH) level in the postmenopausal range may be used to confirm a post-menopausal state in women not using hormonal contraception or hormonal replacement therapy. In the absence of 12 months of amenorrhea, a single FSH measurement is insufficient.);

OR

(2) have had a hysterectomy and/or bilateral oophorectomy, bilateral salpingectomy or bilateral tubal ligation/occlusion, at least 6 weeks prior to screening;

OR

(3) has a congenital or acquired condition that prevents childbearing.

Female and male subjects of reproductive potential must agree to avoid becoming pregnant or impregnating a partner, respectively, while receiving study drug and for 120 days after the last dose of study drug by complying with one of the following:

(1) practice abstinence[†] from heterosexual activity;

OR

(2) use (or have their partner use) acceptable contraception during heterosexual activity.

Acceptable methods of contraception are‡:

Single method (one of the following is acceptable):

- intrauterine device (IUD)
- vasectomy of a female subject's male partner
- contraceptive rod implanted into the skin

Combination method (requires use of two of the following):

- diaphragm with spermicide (cannot be used in conjunction with cervical cap/spermicide)
- cervical cap with spermicide (nulliparous women only)
- contraceptive sponge (nulliparous women only)
- male condom or female condom (cannot be used together)
- hormonal contraceptive: oral contraceptive pill (estrogen/progestin pill or progestin-only pill), contraceptive skin patch, vaginal contraceptive ring, or subcutaneous contraceptive injection

†Abstinence (relative to heterosexual activity) can be used as the sole method of contraception if it is consistently employed as the subject's preferred and usual lifestyle and if considered acceptable by local regulatory agencies and ERCs/IRBs. Periodic abstinence (e.g., calendar, ovulation, sympto-thermal, post-ovulation methods, etc.) and withdrawal are not acceptable methods of contraception.

‡If a contraceptive method listed above is restricted by local regulations/guidelines, then it does not qualify as an acceptable method of contraception for subjects participating at sites in this country/region.

Subjects should be informed that taking the study medication may involve unknown risks to the fetus (unborn baby) if pregnancy were to occur during the study. In order to participate in

the study subjects of childbearing potential must adhere to the contraception requirement (described above) from the day of study medication initiation (or 14 days prior to the initiation of study medication for oral contraception) throughout the study period up to 120 days after the last dose of trial therapy. If there is any question that a subject of childbearing potential will not reliably comply with the requirements for contraception, that subject should not be entered into the study.

8.5.3 Use in Pregnancy

If a subject inadvertently becomes pregnant while on treatment with pembrolizumab, the subject will immediately be removed from the study. The site will contact the subject at least monthly and document the subject's status until the pregnancy has been completed or terminated. The outcome of the pregnancy will be reported to the Merck without delay and within 24 hours to the Merck and within 2 working days to Merck if the outcome is a serious adverse experience (e.g., death, abortion, congenital anomaly, or other disabling or life-threatening complication to the mother or newborn).

The study investigator will make every effort to obtain permission to follow the outcome of the pregnancy and report the condition of the fetus or newborn to the Merck. If a male subject impregnates his female partner the study personnel at the site must be informed immediately and the pregnancy reported to Merck and followed as described above and in Section 7.2.2.

8.5.4 Use in Nursing Women

It is unknown whether pembrolizumab is excreted in human milk. Since many drugs are excreted in human milk, and because of the potential for serious adverse reactions in the nursing infant, subjects who are breast-feeding are not eligible for enrollment.

8.6 Subject Withdrawal/Discontinuation Criteria

Subjects may withdraw consent at any time for any reason or be dropped from the trial at the discretion of the investigator should any untoward effect occur. In addition, a subject may be withdrawn by the investigator if enrollment into the trial is inappropriate, the trial plan is violated, or for administrative and/or other safety reasons. Specific details regarding discontinuation or withdrawal are provided in Section 7.1.4 – Other Procedures.

A subject must be discontinued from the trial for any of the following reasons:

- The subject or legal representative (such as a parent or legal guardian) withdraws consent.

- Confirmed radiographic disease progression

  Note: For unconfirmed radiographic disease progression, please see Section 5.2.2

  Note: A subject may be granted an exception to continue on treatment with confirmed radiographic progression if clinically stable or clinically improved, please see Section 7.1.2.7.1

- Unacceptable adverse experiences as described in Section 5.2.1.2
- Intercurrent illness that prevents further administration of treatment
- Investigator’s decision to withdraw the subject
- The subject has a confirmed positive serum pregnancy test
- Noncompliance with trial treatment or procedure requirements
- The subject is lost to follow-up
- Completed 24 months of uninterrupted treatment with pembrolizumab or 35 administrations of study medication, whichever is later.

  Note: 24 months of study medication is calculated from the date of first dose. Subjects who stop pembrolizumab after 24 months may be eligible for up to one year of additional study treatment if they progress after stopping study treatment provided they meet the requirements detailed in Section 7.1.5.5

- Administrative reasons

The End of Treatment and Follow-up visit procedures are listed in Section 6 (Protocol Flow Chart) and Section 7.1.5 (Visit Requirements). After the end of treatment, each subject will be followed for 30 days for adverse event monitoring (serious adverse events will be collected for 90 days after the end of treatment as described in Section 7.2.3.1). Subjects who discontinue for reasons other than progressive disease will have post-treatment follow-up for disease status until disease progression, initiating a non-study cancer treatment, withdrawing consent or becoming lost to follow-up. After documented disease progression each subject will be followed by telephone for overall survival until death, withdrawal of consent, or the end of the study, whichever occurs first.

8.6.1 Discontinuation of Study Therapy after CR

Discontinuation of treatment may be considered for subjects who have attained a confirmed CR that have been treated for at least 24 weeks with pembrolizumab and had at least two treatments with pembrolizumab beyond the date when the initial CR was declared. Subjects who then experience radiographic disease progression may be eligible for up to one year of additional treatment with pembrolizumab via the Second Course Phase at the discretion of the investigator if no cancer treatment was administered since the last dose of pembrolizumab, the subject meets the safety parameters listed in the Inclusion/Exclusion criteria, and the trial is open. Subjects will resume therapy at the same dose and schedule at the time of initial discontinuation. Additional details are provided in Section 7.1.5.5.

8.7 Subject Replacement Strategy

A subject who discontinues from the trial will not be replaced.

8.8 Clinical Criteria for Early Trial Termination

Early trial termination will be the result of the criteria specified below:

1. Quality or quantity of data recording is inaccurate or incomplete

2. Poor adherence to protocol and regulatory requirements

3. Incidence or severity of adverse drug reaction in this or other studies indicates a potential health hazard to subjects

4. Plans to modify or discontinue the development of the study drug

In the event of Merck decision to no longer supply study drug, ample notification will be provided so that appropriate adjustments to subject treatment can be made.

9.0 TRIAL FLOW CHART

9.1 Study Flow Chart

Trial Period:	Screening Phase	Treatment Cycles [a]						End of Treatment	Post-Treatment		
Treatment Cycle/Title:	Screening (Visit 1)	1	2	3	4	To be repeated beyond 6 cycles<br>5	6	Discon	Safety Follow-up	Follow Up Visits [b]	Survival Follow Up [c]
								At time of discon	30 days post discon	Every 6 weeks post discon	Every 12 weeks
Scheduling Window (Days) [d]:	-28 to -1		± 3	± 3	± 3	± 3	± 3	± 3	± 7	± 7	± 7
**Administrative Procedures**											
Informed Consent [e]	X										
Inclusion/Exclusion Criteria	X										
Demographics and Medical History	X										
Prior and Concomitant Medication Review [f]	X	X	X	X	X	X	X	X	X		
**Clinical Procedures/Assessments**											
Review Adverse Events [g]	X	X	X	X	X	X	X	X	X	X	
12-Lead ECG	X										
Physical Examination	X	X	X	X	X	X	X	X			
Height, Weight, and Vital Signs (T, P, RR, BP) [h]	X	X	X	X	X	X	X	X			
ECOG Performance Status	X	X	X	X	X	X	X	X			
Post-study Anticancer Therapy Status										X	X
Survival Status											X
Trial Treatment Administration											
Pembrolizumab [i]		X	X	X	X	X	X				
**Laboratory Procedures/Assessments:**											
Pregnancy Test – Urine or Serum β-HCG	X										
PT/INR and aPTT [k]	X										
CBC with Differential [l]	X		X	X	X	X	X[l]	X	X[l]		

Trial Period:	Screening Phase	Treatment Cycles [a]						End of Treatment	Post-Treatment		
Treatment Cycle/Title:	Screening (Visit 1)	1	2	3	4	To be repeated beyond 6 cycles<br>5	6	Discon	Safety Follow-up	Follow Up Visits [b]	Survival Follow Up [c]
								At time of discon	30 days post discon	Every 6 weeks post discon	Every 12 weeks
Scheduling Window (Days) [d]:	-28 to -1		± 3	± 3	± 3	± 3	± 3	± 3	± 7	± 7	± 7
Chemistry Panel [l]	X		X	X	X	X	X[l]	X	X[l]		
Urinalysis [l]	X										
T3, FT4 and TSH [l]	X		X		X		X		X		
Hepatitis Lab, Serum AFP, PIVKA-II [l]	X		X		X		X[l]				
Blood for Genetics [m]		X*									
Correlative Blood Samples (DNA) [n]		X*									
Correlative Blood Samples (RNA) [n]		X*	X	X				X			
Correlative Blood Samples (plasma for ctDNA		X*									
Correlative Blood Samples (serum) [n]		X*		X				X			
**Efficacy Measurements**											
Tumor Imaging [o]	X		X		X		X	X[p]		X	
**Tumor Tissue Collection**											
Archival and/or Newly-Obtained Tissue Collection	X	---------------------------X[q]-------------------------------------						X[q]			

*Denotes baseline (-7 day before treatment)

a. Unless otherwise specified, assessments/procedures are to be performed on Day 1 and prior to the first dose of treatment for each cycle.

b. In subjects who discontinued study therapy without documented disease progression, every effort should be made to continue monitoring their disease status by radiologic imaging every 6 weeks (± 7 days) if prior to 1 year or every 9 weeks (± 7 days) if after 1 year, until (1) the start of new anti-cancer treatment, (2) disease progression as assessed by imaging , (3) death, or (4) the end of the study, whichever occurs first.

c. After the start of new anti-cancer treatment or documented disease progression by the imaging , the subject should be contacted by telephone every 12 weeks to assess for survival status. Note: All efforts should be made to ensure telephone contact occurs at least 90 days post discontinue to capture SAEs

d. Unless otherwise specified, the window for each visit is ± 3 days. Cycle 1 treatment must be given within 3 days of enrollment.

e. Written consent must be obtained prior to performing any protocol specified procedure. Results of a test performed prior to the subject signing consent as part of routine clinical management are acceptable in lieu of a screening test if performed within the specified time frame (e.g., within 28 days prior to the first dose of trial treatment).

f. Prior medications – Record all medications taken within 28 days of screening visit. Concomitant medications – Enter new medications started during the trial through the Safety Follow-up visit. Record all medications taken for SAEs

g. Record all AEs occurring within 30 days after the last dose of trial treatment. Report all SAEs (related and unrelated to trial treatment) and ECIs occurring up until 90 days after the last dose of trial treatment or the start of new anti-cancer treatment, whichever comes first. Afterwards, report only SAEs and ECIs that are related to trial treatment.

h. Height will be measured at Visit 1 only. Vitals Signs include temperature, pulse, respiratory rate, and blood pressure.

i. Pembrolizumab should be administered on Day 1 of each three week cycle after all procedures/assessments have been completed.

j. For women of reproductive potential, a serum pregnancy test should be performed within 72 hours prior to first dose of trial treatment. A urine test can be considered if serum is not appropriate. Pregnancy tests (serum and/or urine tests) should be repeated if required by local guidelines.

k. Coagulation factors (PT/INR and aPTT) should be tested as part of the screening procedures for all subjects.

l Laboratory tests for screening are to be performed within 10 days prior to the first dose of trial treatment.
After Cycle1, lab samples can be collected up to 72 hours prior to the scheduled time point. To be repeated every 2 cycles after Cycle 6. Unresolved abnormal labs that are drug relatedAEs should be followed until resolution. Labs do not need to be repeated after the end of treatment if labs are within normal range.
At screening, Hepatitis labs include HepB surface antigen, HepB surface antibody, Hep B Core antibody, HepB DNA Viralload (PCR), Hep C viral load (PCR) and Hep C Antibody, Hep D antibody. During treatment, hepatitis labs will be conducted as follows ; For HCV infected--HCV RNA every 6 weeks on treatment (every other cycle) through cycle 8, then every 6 cycles thereafter.
For HBV infected--HBV DNA every 6 weeks on treatment

m. This sample should be drawn for planned genetic analysis of DNA and drug response unless there is either a documented law or regulation prohibiting collection, or unless the IRB/IEC does not approve of the collection of the sample for these purposes.

n. Whole blood sample for correlative studies should be collected at Cycle 1, Day 1- Pre-dose, Cycle 2 Day 1- Pre-dose, Cycle 3 Day 1 Pre-dose and again at treatment discontinuation. Blood for serum and blood for plasma to be collected only prior to Cycle 1 Day 1.

o. Baseline tumor imaging will be performed within 14 days prior to enrollment. Scans performed as part of routine clinical management are acceptable for use as the baseline scan if they are of diagnostic quality . The exact same image acquisition and processing parameters should be used throughout the study. The first on-study imaging time point will be performed 6 weeks (± 7 days) or earlier if clinically indicated and will continue to be performed every 6 weeks (± 7 days) regardless of any treatment delays. Following week 24 (6 months), imaging time point will occur every 12 weeks (± 7 days) while the subject remain on trial. Imaging timing should follow calendar days. On-study scans should be submitted immediately to the imaging .

p. In subjects who discontinue study therapy without verified disease progression, a radiologic evaluation should be performed at the time of treatment discontinuation (i.e., date of discontinuation ± 4 week window). If a previous scan was obtained within 4 weeks prior to the date of discontinuation, then a scan at treatment discontinuation is not required.

q. An optional newly-obtained core or excisional biopsy (FNA not adequate) is requested at any post-treatment time point during the study, (preference would be as close to dosing at Week 12 as possible). A biopsy is also requested at the time of discontinuation for PD, but will not be required. Endoscopic biopsies are permitted.

10.0 TRIAL PROCEDURES

10.1 Trial Procedures

The Trial Flow Chart summarizes the trial procedures to be performed at each visit. Individual trial procedures are described in detail below. It may be necessary to perform these procedures at unscheduled time points if deemed clinically necessary by the investigator.

10.1.1 Administrative Procedures

10.1.1.1 Informed Consent

The Investigator must obtain documented consent from each potential subject prior to participating in a clinical trial.

10.1.1.2 General Informed Consent

Consent must be documented by the subject's dated signature or by the subject's legally acceptable representative's dated signature on a consent form along with the dated signature of the person conducting the consent discussion.

A copy of the signed and dated consent form should be given to the subject before participation in the trial.

The initial informed consent form, any subsequent revised written informed consent form and any written information provided to the subject must receive the IRB approval/favorable opinion in advance of use. The subject or his/her legally acceptable representative should be informed in a timely manner if new information becomes available that may be relevant to the subject's willingness to continue participation in the trial. The communication of this information will be provided and documented via a revised consent form or addendum to the original consent form that captures the subject's dated signature or by the subject's legally acceptable representative's dated signature.

The informed consent will adhere to IRB requirements, applicable laws and regulations

10.1.1.3 Inclusion/Exclusion Criteria

All inclusion and exclusion criteria will be reviewed by the investigator or qualified designee to ensure that the subject qualifies for the trial.

10.1.1.4 Medical History

A medical history will be obtained by the investigator or qualified designee. Medical history will include all active conditions, and any condition diagnosed within the prior 10 years that are considered to be clinically significant by the Investigator. Details regarding the disease for which the subject has enrolled in this study will be recorded separately and not listed as medical history.

10.1.1.5 Prior and Concomitant Medications Review

10.1.1.5.1 Prior Medications

The investigator or qualified designee will review prior medication use, including any protocol-specified washout requirement, and record prior medication taken by the subject within 28 days before starting the trial. Treatment for the disease for which the subject has enrolled in this study will be recorded separately and not listed as a prior medication.

10.1.1.5.2 Concomitant Medications

The investigator or qualified designee will record medication, if any, taken by the subject during the trial. All medications related to reportable SAEs should be recorded.

10.1.1.6 Disease Details and Treatments

10.1.1.6.1 Disease Details

The investigator or qualified designee will obtain prior and current details regarding disease status.

10.1.1.6.2 Prior Treatment Details

The investigator or qualified designee will review all prior cancer treatments including systemic treatments, radiation and surgeries.

10.1.1.6.3 Subsequent Anti-Cancer Therapy Status

The investigator or qualified designee will review all new anti-neoplastic therapy initiated after the last dose of trial treatment. If a subject initiates a new anti-cancer therapy within 30 days after the last dose of trial treatment, the 30 day Safety Follow-up visit must occur before the first dose of the new therapy. Once new anti-cancer therapy has been initiated the subject will move into survival follow-up.

10.1.1.7 Assignment of Screening Number

All consented subjects will be given a unique screening number that will be used to identify the subject for all procedures that occur prior to enrollment. Each subject will be assigned only one screening number. Screening numbers must not be re-used for different subjects.

Any subject who is screened multiple times will retain the original screening number assigned at the initial screening visit.

10.1.1.8 Assignment of Enrollment Number

All eligible subjects will be enrollment and will receive a enrollment number.

10.1.1.9 Trial Compliance (Medication/Diet/Activity/Other)

Interruptions from the protocol specified treatment plan for greater than 12 weeks between pembrolizumab doses require consultation between the investigator and written documentation of the collaborative decision on subject management.

Administration of trial medication will be witnessed by the investigator and/or trial staff.

10.1.2 Clinical Procedures/Assessments

10.1.2.1 Adverse Event (AE) Monitoring

The investigator or qualified designee will assess each subject to evaluate for potential new or worsening AEs as specified in the Trial Flow Chart and more frequently if clinically indicated. Adverse experiences will be graded and recorded throughout the study and during the follow-up period according to NCI CTCAE Version 4.0 .Toxicities will be characterized in terms regarding seriousness, causality, toxicity grading, and action taken with regard to trial treatment.

10.1.2.2 Physical Exam

The investigator or qualified designee will perform a physical exam during the study period. Clinically significant abnormal findings should be recorded as medical history.

10.1.2.3 Vital Signs

The investigator or qualified designee will take vital signs at screening, prior to the administration of each dose of trial treatment and at treatment discontinuation as specified in the Trial Flow Chart (Section 6.0). Vital signs should include temperature, pulse, respiratory rate, weight and blood pressure. Height will be measured at screening only.

10.1.2.4 Eastern Cooperative Oncology Group (ECOG) Performance Scale

The investigator or qualified designee will assess ECOG status (see Section 18.1) at screening, prior to the administration of each dose of trial treatment and discontinuation of trial treatment as specified in the Trial Flow Chart.

10.1.2.5 Tumor Tissue Collection and Correlative Studies Blood Sampling

Participation in this trial will be dependent upon supplying a tumor tissue specimen. Newly obtained endoscopic biopsy specimens are preferred to fresh samplses, archived samples and formalin -fixed, paraffin-embedded (FFPE) block specimens are preferred to slides.

Note: A fine needle aspirate (FNA) or cytologic specimen will not be acceptable. Newly obtained endoscopic biopsy specimen or archived tissue should be submitted. If there is an existing specimen obtained with surgical resection or core needle biopsy, these can be submitted. Newly-obtained specimens are defined as FFPE-preserved blocks of tissue collected up to 12 weeks prior to Day 1.

10.1.3 Laboratory Procedures/Assessments

Details regarding specific laboratory procedures/assessments to be performed in this trial are provided belowLaboratory Safety Evaluations (Hematology, Chemistry and Urinalysis)

Laboratory tests for hematology, chemistry, urinalysis, and others are specified in Table 5.

Table 5 Laboratory Tests

Hematology	Chemistry	Urinalysis	Other
Hematocrit	Albumin	Blood	Serum β-human chorionic gonadotropin†
Hemoglobin	Alkaline phosphatase	Glucose	(β-hCG)†
Platelet count	Alanine aminotransferase (ALT)	Protein	PT (INR)
WBC (total and differential)	Aspartate aminotransferase (AST)	Specific gravity	aPTT
Red Blood Cell Count	Lactate dehydrogenase (LDH)	Microscopic exam *(If abnormal)*	Total thriiodothyronine (T3)
Absolute Neutrophil Count	Uric Acid	results are noted	Free tyroxine (T4)
Absolute Lymphocyte Count	Calcium	Urine pregnancy test †	Thyroid stimulating hormone (TSH)
	Chloride		
	Glucose		Blood for correlative studies
	Phosphorus		HBV
	Potassium		HCV
	Sodium		
	Magnesium		
	Total Bilirubin		
	Direct Bilirubin *(If total bilirubin is elevated above the upper limit of normal)*		
	Total protein		
	Blood Urea Nitrogen		
† Perform on women of childbearing potential only. If urine pregnancy results cannot be confirmed as negative, a serum pregnancy test will be required.			

Laboratory tests for screening or entry into the Second Course Phase should be performed within 10 days prior to the first dose of treatment. After Cycle 1, pre-dose laboratory procedures can be conducted up to 72 hours prior to dosing. Results must be reviewed by the investigator or qualified designee and found to be acceptable prior to each dose of trial treatment.

10.1.4 Other Procedures

10.1.4.1 Withdrawal/Discontinuation

When a subject discontinues/withdraws prior to trial completion, all applicable activities scheduled for the final trial visit should be performed at the time of discontinuation. Any adverse events which are present at the time of discontinuation/withdrawal should be followed in accordance with the safety requirements outlined - Assessing and Recording Adverse Events. Subjects who a) attain a CR or b) complete 24 months of treatment with pembrolizumab may discontinue treatment with the option of restarting treatment if they meet the criteria specified. After discontinuing treatment following assessment of CR, these subjects should return to the site for a Safety Follow-up Visit and then proceed to the Follow-Up Period of the study

10.1.5 Visit Requirements

Visit requirements are outlined in Section 9.0 - Trial Flow Chart. Specific procedure-related details are provided above in Section 10.0 - Trial Procedures.

10.1.5.1 Screening

10.1.5.1.1 Screening Period

Approximately 28 days prior to enrollment, potential subjects will be evaluated to determine that they fulfill the entry requirements as set forth in Section 8.1.
Results of a test performed prior to the subject signing consent as part of routine clinical management are acceptable in lieu of a screening test if performed within the specified time frame. Screening procedures are to be completed within 28 days prior to the first dose trial treatment except for the following:

- Laboratory tests and ECOG PS are to be performed within 10 days prior to the first dose of trial treatment.
- For women of reproductive potential, a serum pregnancy test will be performed within 72 hours prior to the first dose of trial treatment. A urine test may be considered if serum test is not appropriate.
- Baseline tumor imaging will be performed within 14 days prior to enrollment for all subjects. Scans performed as part of routine clinical management are acceptable for use as the baseline scan

10.1.5.2 Treatment Period

Visit requirements are outlined in Section 9.0

10.1.5.3 Discontinuation Visit

The Discontinuation Visit should occur at the time study treatment is discontin ued for any reason. If the Discontinuation Visit occurs 30 days from the last dose of study treatment, at the time of the mandatory Safety Follow up Visit, procedures do not need to be repeated. Visit requirements are outlined in Section 9.0

10.1.5.4 Post-Treatment Visits

10.1.5.4.1 Safety Follow-Up Visit

The mandatory Safety Follow-Up Visit should be conducted approximately 30 days after the last dose of trial treatment or before the initiation of a new anti-cancer treatment, whichever comes first. All AEs that occur prior to the Safety Follow-Up Visit should be recorded. Subjects with an AE of Grade > 1 will be followed until the resolution of the AE to Grade 0-1 or until the beginning of a new anti-neoplastic therapy, whichever occurs first. SAEs that occur within 90 days of the end of treatment or before initiation of a new anti-cancer treatment should also be followed and recorded.

10.1.5.4.2 Follow-up Visits

Subjects who discontinue trial treatment for a reason other than disease progression will move into the Follow-Up Phase and should be assessed every 6 weeks (42 ± 7 days) by radiologic imaging to monitor disease status. After 1 year, the imaging time point will occur every 9 weeks (± 7 days). Every effort should be made to collect information regarding disease status until the start of new anti-neoplastic therapy, disease progression, death, end of the study .Information regarding post-study anti-neoplastic treatment will be collected if new treatment is initiated.

10.1.5.4.3 Survival Follow-up

Once a subject experiences confirmed disease progression or starts a new anti-cancer therapy, the subject moves into the survival follow-up phase and should be contacted by telephone every 12 weeks to assess for survival status until death, withdrawal of consent, or the end of the study, whichever occurs first.

10.2 Assessing and Recording Adverse Events

An adverse event is defined as any untoward medical occurrence in a patient or clinical investigation subject administered a pharmaceutical product and which does not necessarily have to have a causal relationship with this treatment. An adverse event can therefore be any unfavorable and unintended sign (including an abnormal laboratory finding, for example), symptom, or disease temporally associated with the use of a medicinal product or protocol-specified procedure, whether or not considered related to the medicinal product or protocol-specified procedure. Any worsening (i.e., any clinically significant adverse change in frequency and/or intensity) of a preexisting condition that is temporally associated with the use of the Merck's product, is also an adverse event.

Changes resulting from normal growth and development that do not vary significantly in frequency or severity from expected levels are not to be considered adverse events. Examples of this may include, but are not limited to, teething, typical crying in infants and children and onset of menses or menopause occurring at a physiologically appropriate time.

Merck product includes any pharmaceutical product, biological product, device, diagnostic agent or protocol-specified procedure, whether investigational (including placebo or active comparator medication) or marketed, manufactured by, licensed by, provided by or distributed by Merck for human use.

Adverse events may occur during the course of the use of Merck product in clinical trials or as prescribed in clinical practice, from overdose (whether accidental or intentional), from abuse and from withdrawal.

Progression of the cancer under study is not considered an adverse event.

All adverse events that occur after the consent form is signed but before treatment allocation/randomization must be reported by the investigator if they cause the subject to be excluded from the trial, or are the result of a protocol-specified intervention, including but not limited to washout or discontinuation of usual therapy, diet, placebo treatment or a procedure.

From the time of treatment allocation/randomization through 30 days following cessation of treatment, all adverse events must be reported by the investigator. Such events will be recorded at each examination on the Adverse Event case report forms/worksheets. The reporting timeframe for adverse events meeting any serious criteria is described in section 7.2.3.1. The investigator will make every attempt to follow all subjects with non-serious adverse events for outcome.

Adverse events will not be collected for subjects during the pre-screening period (for determination of archival tissue status) as long as that subject has not undergone any protocol-specified procedure or intervention. If the subject requires a blood draw, fresh tumor biopsy etc., the subject is first required to provide consent to the main study and AEs will be captured according to guidelines for standard AE reporting.

10.2.1 Definition of an Overdose for This Protocol and Reporting of Overdose to Merck

For purposes of this trial, an overdose of pembrolizumab will be defined as any dose of 1,000 mg or greater (≥5 times the indicated dose). No specific information is available on the treatment of overdose of pembrolizumab. Appropriate supportive treatment should be provided if clinically indicated. In the event of overdose, the subject should be observed closely for signs of toxicity. Appropriate supportive treatment should be provided if clinically indicated.

If an adverse event(s) is associated with ("results from") the overdose of a Merck product, the adverse event(s) is reported as a serious adverse event, even if no other seriousness criteria are met.

If a dose of Merck's product meeting the protocol definition of overdose is taken without any associated clinical symptoms or abnormal laboratory results, the overdose is reported as a non-

serious Event of Clinical Interest (ECI), using the terminology "accidental or intentional overdose without adverse effect."

All reports of overdose with and without an adverse event must be reported within 24 hours to the IRB and within 2 working days hours to Merck Global Safety. (Attn: Worldwide Product Safety; FAX 215 993-1220)

10.2.2 Reporting of Pregnancy and Lactation to Merck

Although pregnancy and lactation are not considered adverse events, it is the responsibility of investigators or their designees to report any pregnancy or lactation in a subject (spontaneously reported to them) that occurs during the trial.

Pregnancies and lactations that occur after the consent form is signed but before treatment allocation/randomization must be reported by the investigator if they cause the subject to be excluded from the trial, or are the result of a protocol-specified intervention, including but not limited to washout or discontinuation of usual therapy, diet, placebo treatment or a procedure.

Pregnancies and lactations that occur from the time of treatment allocation/randomization through 120 days following cessation of Sponsor's product, or 30 days following cessation of treatment if the subject initiates new anticancer therapy, whichever is earlier, must be reported by the investigator. All reported pregnancies must be followed to the completion/termination of the pregnancy. Pregnancy outcomes of spontaneous abortion, missed abortion, benign hydatidiform mole, blighted ovum, fetal death, intrauterine death, miscarriage and stillbirth must be reported as serious events (Important Medical Events). If the pregnancy continues to term, the outcome (health of infant) must also be reported.

Such events must be reported within 24 hours to the IRB and within 2 working days to Merck Global Safety. (Attn: Worldwide Product Safety; FAX 215 993-1220)

10.2.3 Immediate Reporting of Adverse Events to Merck

10.2.3.1 Serious Adverse Events

A serious adverse event is any adverse event occurring at any dose or during any use of Merck's product that:

- Results in death;
- Is life threatening;
- Results in persistent or significant disability/incapacity;
- Results in or prolongs an existing inpatient hospitalization;
- Is a congenital anomaly/birth defect;
- Is an other important medical event

- **Note:** In addition to the above criteria, adverse events meeting either of the below criteria, although not serious per ICH definition, are reportable to the Merck in the same timeframe as SAEs to meet certain local requirements. Therefore, these events are considered serious by Merck for collection purposes.

- Is a new cancer (that is not a condition of the study);
- Is associated with an overdose.

Refer to Table 6 for additional details regarding each of the above criteria.

For the time period beginning when the consent form is signed until treatment allocation/randomization, any serious adverse event, or follow up to a serious adverse event, including death due to any cause other than progression of the cancer under study (reference Section 7.2.3.3 for additional details) that occurs to any subject must be reported within 24 hours to the Sponsor and within 2 working days to Merck Global Safety if it causes the subject to be excluded from the trial, or is the result of a protocol-specified intervention, including but not limited to washout or discontinuation of usual therapy, diet, placebo treatment or a procedure.

For the time period beginning at treatment allocation/randomization through 90 days following cessation of treatment, or 30 days following cessation of treatment if the subject initiates new anticancer therapy, whichever is earlier, any serious adverse event, or follow up to a serious adverse event, including death due to any cause other than progression of the cancer under study (reference Section 7.2.3.3 for additional details), whether or not related to the Merck product, must be reported within 24 hours to the Sponsor and within 2 working days to Merck Global Safety.

Additionally, any serious adverse event, considered by an investigator who is a qualified physician to be related to Merck product that is brought to the attention of the investigator at any time following consent through the end of the specified safety follow-up period specified in the paragraph above, or at any time outside of the time period specified in the previous paragraph also must be reported immediately to the Sponsor and to Merck Global Safety.

All subjects with serious adverse events must be followed up for outcome.

SAE reports and any other relevant safety information are to be forwarded to the Merck Global Safety facsimile number: +1-215-993-1220

A copy of all 15 Day Reports and Annual Progress Reports is submitted as required by FDA, European Union (EU), Pharmaceutical and Medical Devices agency (PMDA) or other local regulators. Investigators will cross reference this submission according to local regulations to the Merck Investigational Compound Number (IND, CSA, etc.) at the time of submission. Additionally investigators will submit a copy of these reports to Merck & Co., Inc. (Attn: Worldwide Product Safety; FAX 215 993-1220) at the time of submission to FDA.

10.2.3.2 Events of Clinical Interest

Selected non-serious and serious adverse events are also known as Events of Clinical Interest (ECI) and must be reported within 24 hours to IRB and within 2 working days to Merck Global Safety. (Attn: Worldwide Product Safety; FAX 215 993-1220)

For the time period beginning when the consent form is signed until treatment allocation/randomization, any ECI, or follow up to an ECI, that occurs to any subject must be reported within 24 hours to the Sponsor and within 2 working days to Merck Global Safety if it causes the subject to be excluded from the trial, or is the result of a protocol-specified intervention, including but not limited to washout or discontinuation of usual therapy, diet, placebo treatment or a procedure.

For the time period beginning at treatment allocation/randomization through 90 days following cessation of treatment, or 30 days following cessation of treatment if the subject initiates new anticancer therapy, whichever is earlier, any ECI, or follow up to an ECI, whether or not related to Merck product, must be reported within 24 hours to the Sponsor and within 24 hours to Merck Global Safety.

Events of clinical interest for this trial include:

1. an overdose of Merck product, as defined in Section 10.2.1 - Definition of an Overdose for This Protocol and Reporting of Overdose to Merk, that is not associated with clinical symptoms or abnormal laboratory results.

2. an elevated AST or ALT lab value that is greater than or equal to 3X the upper limit of normal and an elevated total bilirubin lab value that is greater than or equal to 2X the upper limit of normal and, at the same time, an alkaline phosphatase lab value that is less than 2X the upper limit of normal, as determined by way of protocol-specified laboratory testing or unscheduled laboratory testing.*

> *Note: These criteria are based upon available regulatory guidance documents. The purpose of the criteria is to specify a threshold of abnormal hepatic tests that may require an additional evaluation for an underlying etiology.

10.2.3.3 Protocol-Specific Exceptions to Serious Adverse Event Reporting

Efficacy endpoints as outlined in this section will not be reported to Merck as described in Section 7.2.3.- Immediate Reporting of Adverse Events to the Sponsor and to Merck, unless there is evidence suggesting a causal relationship between the drug and the event. Any such event will be submitted to the Sponsor within 24 hours and to Merck Global Safety within 2 working days either by electronic or paper media.

Specifically, the suspected/actual events covered in this exception include any event that is disease progression of the cancer under study.

The Sponsor will monitor unblinded aggregated efficacy endpoint events and safety data to ensure the safety of the subjects in the trial. Any suspected endpoint which upon review is not progression of the cancer under study will be forwarded to Merck Global Safety as a SAE within 2 working days of determination that the event is not progression of the cancer under study

Hospitalization related to convenience (e.g.transportation issues etc.) will not be considered a SAE.

10.2.4 Evaluating Adverse Events

An investigator who is a qualified physician will evaluate all adverse events according to the NCI Common Terminology for Adverse Events (CTCAE), version 4.0. Any adverse event which changes CTCAE grade over the course of a given episode will have each change of grade recorded on the adverse event case report forms/worksheets.

All adverse events regardless of CTCAE grade must also be evaluated for seriousness.

Table 6 Evaluating Adverse Events

An investigator who is a qualified physician, will evaluate all adverse events as to:

V4.0 CTCAE Grading	**Grade 1**	Mild; asymptomatic or mid symptoms; clinical or diagnostic observations only; intervention not indicated.
	**Grade 2**	Moderate; minimal, local or noninvasive intervention indicated; limiting age-appropriate instrumental ADL.
	**Grade 3**	Severe or medically significant but not immediately life-threatening; hospitalization or prolongation or hospitalization indicated; disabling; limiting self-care ADL.
	**Grade 4**	Life threatening consequences; urgent intervention indicated.
	**Grade 5**	Death related to AE
**Seriousness**	A serious adverse event is any adverse event occurring at any dose or during any use of Merck product that:	
	†**Results in death**; or	
	†**Is life threatening; or** places the subject, in the view of the investigator, at immediate risk of death from the event as it occurred (Note: This does not include an adverse event that, had it occurred in a more severe form, might have caused death.); or	
	†**Results in a persistent or significant disability/incapacity** (substantial disruption of one's ability to conduct normal life functions); or	
	†**Results in or prolongs an existing inpatient hospitalization** (hospitalization is defined as an inpatient admission, regardless of length of stay, even if the hospitalization is a precautionary measure for continued observation. (Note: Hospitalization for an elective procedure to treat a pre-existing condition that has not worsened is not a serious adverse event. A pre-existing condition is a clinical condition that is diagnosed prior to the use of a Merck product and is documented in the patient's medical history.); or	
	†**Is a congenital anomaly/birth defect** (in offspring of subject taking the product regardless of time to diagnosis);or	
	**Is a** new **cancer** (that is not a condition of the study) **(**although not serious per ICH definition, is reportable to the Sponsor within 24 hours and to Merck within 2 working days to meet certain local requirements); **or**	
	**Is an overdose** (whether accidental or intentional). Any adverse event associated with an overdose is considered a serious adverse event for collection purposes. An overdose that is not associated with an adverse event is considered a non-serious event of clinical interest and must be reported within 24 hours to the Sponsor and to Merck within 2 working days..	

	**Other important medical events** that may not result in death, not be life threatening, or not require hospitalization may be considered a serious adverse event when, based upon appropriate medical judgment, the event may jeopardize the subject and may require medical or surgical intervention to prevent one of the outcomes listed previously (designated above by a †).	
**Duration**	Record the start and stop dates of the adverse event. If less than 1 day, indicate the appropriate length of time and units	
**Action taken**	Did the adverse event cause Merck product to be discontinued?	
**Relationship to Merck Product**	Did Merck product cause the adverse event? The determination of the likelihood that Merck product caused the adverse event will be provided by an investigator who is a qualified physician. The investigator's signed/dated initials on the source document or worksheet that supports the causality noted on the AE form, ensures that a medically qualified assessment of causality was done. This initialed document must be retained for the required regulatory time frame. The criteria below are intended as reference guidelines to assist the investigator in assessing the likelihood of a relationship between the test drug and the adverse event based upon the available information.<br><br>**The following components are to be used to assess the relationship between Merck product and the AE**; the greater the correlation with the components and their respective elements (in number and/or intensity), the more likely Merck product caused the adverse event (AE):	
	**Exposure**	Is there evidence that the subject was actually exposed to Merck product such as: reliable history, acceptable compliance assessment (pill count, diary, etc.), expected pharmacologic effect, or measurement of drug/metabolite in bodily specimen?
	**Time Course**	Did the AE follow in a reasonable temporal sequence from administration of Merck product? Is the time of onset of the AE compatible with a drug-induced effect (applies to trials with investigational medicinal product)?
	**Likely Cause**	Is the AE not reasonably explained by another etiology such as underlying disease, other drug(s)/vaccine(s), or other host or environmental factors

**Relationship to Merck Product (continued)**	**The following components are to be used to assess the relationship between the test drug and the AE: (continued)**	
	**Dechallenge**	Was Merck product discontinued or dose/exposure/frequency reduced?<br><br>If yes, did the AE resolve or improve?<br><br>If yes, this is a positive dechallenge. If no, this is a negative dechallenge.<br><br>(Note: This criterion is not applicable if: (1) the AE resulted in death or permanent disability; (2) the AE resolved/improved despite continuation of the Sponsor's product; or (3) the trial is a single-dose drug trial); or (4) Sponsor's product(s) is/are only used one time.)
	**Rechallenge**	Was the subject re-exposed to Merck product in this study?

		If yes, did the AE recur or worsen?<br><br>If yes, this is a positive rechallenge. If no, this is a negative rechallenge.<br><br>(Note: This criterion is not applicable if: (1) the initial AE resulted in death or permanent disability, or (2) the trial is a single-dose drug trial); or (3) Sponsor's product(s) is/are used only one time).<br><br>NOTE: IF A RECHALLENGE IS PLANNED FOR AN ADVERSE EVENT WHICH WAS SERIOUS AND WHICH MAY HAVE BEEN CAUSED BY MERCK PRODUCT, OR IF REEXPOSURE TO MERCK PRODUCT POSES ADDITIONAL POTENTIAL SIGNIFICANT RISK TO THE SUBJECT, THEN THE RECHALLENGE MUST BE APPROVED IN ADVANCE BY THE SPONSOR AS PER DOSE MODIFICATION GUIDELINES IN THE PROTOCOL.
	**Consistency with Trial Treatment Profile**	Is the clinical/pathological presentation of the AE consistent with previous knowledge regarding Merck product or drug class pharmacology or toxicology?
The assessment of relationship will be reported on the case report forms /worksheets by an investigator who is a qualified physician according to his/her best clinical judgment, including consideration of the above elements.		
**Record one of the following**		**Use the following scale of criteria as guidance (not all criteria must be present to be indicative of Merck product relationship).**
**Yes, there is a reasonable possibility of Merck product relationship.**		There is evidence of exposure to Merck product. The temporal sequence of the AE onset relative to the administration of Merck product is reasonable. The AE is more likely explained by Merck product than by another cause.
**No, there is not a reasonable possibility of Merck product relationship**		Subject did not receive the Merck product OR temporal sequence of the AE onset relative to administration of Merck product is not reasonable OR the AE is more likely explained by another cause than the Merck product. (Also entered for a subject with overdose without an associated AE.)

10.2.5 investigators Responsibility for Reporting Adverse Events

All Adverse Events will be reported to regulatory authorities, IRB in accordance with all applicable global laws and regulations.

11.0 STATISTICAL ANALYSIS PLAN

11.1 Statistical Analysis Plan

11.1.1 Efficacy

11.1.1.1 ORR

The intention to treat (ITT) population included all patients who received at least one dose of permbrolizumab. ORR was calculated with two-sided 95% confidence intervals for the overall population. ORR was defined as (CR + PR)/(number of ITT population).

11.1.1.2 Biomarker

To explore and identify biomarkers (inform the scientific understanding of diseases and/or their therapeutic treatment), the contingency tables will be presented by response for each of tumor histology, molecular signatures, immune pathways and etc.

① prospective storage of plasma for ctDNA evaluation (Streck tubes) in all enrolled-patients.

② In the actual Seq, comparison btw responder vs non-responder will be conducted in selected-population using prospectively collected samples.

③ Timepoints of collection : 1) pre-treatment (baseline), 2)prior to 3rd dose (C3), and 3)at progression.

11.1.2 Safety

Safety will be assessed by clinical review of all relevant parameters including, adverse events (AEs), laboratory tests, vital signs, etc.
No statistical hypothesis tests will be performed on safety variables. These will be summarized by descriptive statistics or categorical tables.

11.2 Sample size

About using one-stage design response rate (ORR) hypothesis opposed to expect 5% of the null hypothesis that the response rate of 18.5% or more (two-tailed), the first species of 5%. Assuming about 10% of attrition due to ineligibility and dropout, we will recruit 60 patients

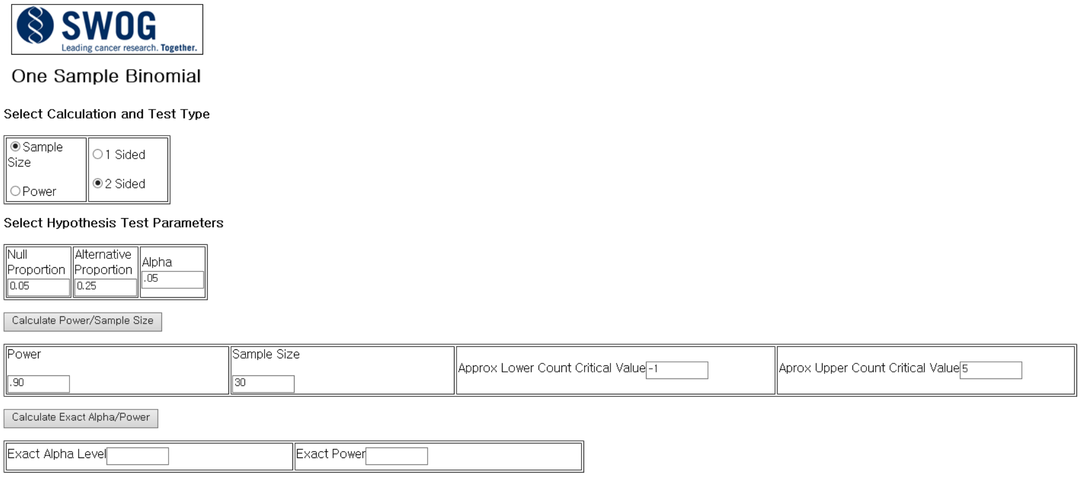
SWOG
Leading cancer research. Together.

One Sample Binomial

Select Calculation and Test Type

Sample Size (selected) | Power | 1 Sided | 2 Sided (selected)

Select Hypothesis Test Parameters

Null Proportion	Alternative Proportion	Alpha
0.05	0.25	.05

Calculate Power/Sample Size

Power	Sample Size	Approx Lower Count Critical Value	Aprox Upper Count Critical Value
.90	30	-1	5

Calculate Exact Alpha/Power

Exact Alpha Level	Exact Power

12.0 LABELING, PACKAGING, STORAGE AND RETURN OF CLINICAL SUPPLIES

12.1 Investigational Product

The investigator shall take responsibility for and shall take all steps to maintain appropriate records and ensure appropriate supply, storage, handling, distribution and usage of investigational product in accordance with the protocol and any applicable laws and regulations.

Clinical Supplies will be provided by Merck as summarized in Table 7.

Table 7 Product Descriptions

Product Name & Potency	Dosage Form
Pembrolizumab 50 mg	Lyophilized Powder for Injection
Pembrolizumab 100 mg/ 4mL	Solution for Injection

12.2 Packaging and Labeling Information

Clinical supplies will be affixed with a clinical label in accordance with regulatory requirements.

12.3 Clinical Supplies Disclosure

This trial is open-label; therefore, the subject, the trial site personnel, designee are not blinded to treatment. Drug identity (name, strength) is included in the label text

12.4 Storage and Handling Requirements

Clinical supplies must be stored in a secure, limited-access location under the storage conditions specified on the label.

Receipt and dispensing of trial medication must be recorded by an authorized person at the trial site.

Clinical supplies may not be used for any purpose other than that stated in the protocol.

12.5 Returns and Reconciliation

The investigator is responsible for keeping accurate records of the clinical supplies received from Merck or designee, the amount dispensed to and returned by the subjects and the amount remaining at the conclusion of the trial.

Upon completion or termination of the study, all unused and/or partially used investigational product will be destroyed at the site per institutional policy. It is the Investigator's responsibility to arrange for disposal of all empty containers, provided that procedures for proper disposal have been established according to applicable federal, state, local and institutional guidelines and procedures, and provided that appropriate records of disposal are kept.

13.0 ADMINISTRATIVE AND REGULATORY DETAILS

13.1 Confidentiality

13.1.1 Confidentiality of Data

By signing this protocol, the investigator affirms to the investigator that information furnished will be maintained in confidence, and such information will be divulged to the institutional review board, ethics review committee (IRB) or similar or expert committee; affiliated institution and employees, only under an appropriate understanding of confidentiality with such board or committee, affiliated institution and employees. Data generated by this trial will be considered confidential by the investigator, except to the extent that it is included in a publication as provided in the Publications section of this protocol.

13.1.2 Confidentiality of Subject Records

By signing this protocol, the investigator agrees that IRB, or regulatory authority representatives may consult trial documents in order to verify worksheet/case report form data. By signing the consent form, the subject agrees to this process. If trial documents will be photocopied during the process of verifying worksheet/case report form information, the subject will be identified by unique code only

By signing this protocol, the investigator agrees to treat all subject data used and disclosed in connection with this trial in accordance with all applicable privacy laws, rules and regulations.

13.2 Compliance with Financial Disclosure Requirements

Not applicable to this study.

13.3 Compliance with Law, Audit and Debarment

13.4 Compliance with Trial Registration and Results Posting Requirements

Under the terms of the Food and Drug Administration Modernization Act (FDAMA) and the Food and Drug Administration Amendments Act (FDAAA), the investigator of the trial is solely responsible for determining whether the trial and its results are subject to the requirements for submission to the Clinical Trials Data Bank, http://www.clinicaltrials.gov. Information posted will allow subjects to identify potentially appropriate trials for their disease conditions and pursue participation by calling a central contact number for further information on appropriate trial locations and trial site contact information.

14.0 MONITORING

A clinical monitoring will make regularly scheduled trips to the investigational site to review the progress of the trial. The actual frequency of monitoring trips will depend on the enrollment rate and performance at each site. The investigator will allow monitor, and/or its representatives of designees, access to all pertinent medical records in order to allow for the verification of data gathered in the CRFs and for the review of the data collection process. At each visit, the monitor will review various aspects of the trial including, but not limited to: screening and enrollment logs; compliance with the protocol and study manual and with the principles of Good Clinical Practice; completion of case report forms; source data verification; study drug accountability and storage; facilities and staff.

15.0 DATA HANDLING

To enable evaluations and/or audits from regulatory authorities, the Investigator agrees to keep records, including the identity of all participating subjects (sufficient information to link records, eg, CRFs and hospital records), all original signed informed consent forms, copies of all CRFs, source documents, and detailed records of treatment disposition. The records should be retained by the Investigator according to ICH, local regulations, or as specified in the Clinical Study Agreement, whichever is longer.

16.0 SAMPLE IDENTIFICATION AND RETENSION SPECIMEN

All subjects information will be anonymized. A ‘Sample ID’ number will be recorded.
Upon expiration of the retention period determined by subject, the human-derived material shall be destroyed in accordance with the standards and methods per the [Wastes Control Act] Article 13.

17.0 REFERENCES

1. Jemal A, Bray F, Center MM, Ferlay J, Ward E, Forman D. Global cancer statistics. CA Cancer J Clin 2011;61:69-90.

2. Kirk GD, Lesi OA, Mendy M, Akano AO, Sam O, Goedert JJ, et al. The Gambia Liver Cancer Study: Infection with hepatitis B and C and the risk of hepatocellular carcinoma in West Africa. Hepatology 2004;39:211-9.

3. Hung H. Treatment modalities for hepatocellular carcinoma. Curr Cancer Drug Targets 2005;5:131-8.

4. Bruix J, Sherman M. Management of hepatocellular carcinoma. Hepatology 2005;42:1208-36.

5. Llovet JM, Ricci S, Mazzaferro V, Hilgard P, Gane E, Blanc JF, et al. Sorafenib in advanced hepatocellular carcinoma. N Engl J Med 2008;359:378-90.

6. Cheng AL, Kang YK, Chen Z, Tsao CJ, Qin S, Kim JS, et al. Efficacy and safety of sorafenib in patients in the Asia-Pacific region with advanced hepatocellular carcinoma: a phase III randomised, double-blind, placebo-controlled trial. Lancet Oncol 2009;10:25-34.

7. Wilhelm SM, Carter C, Tang L, Wilkie D, McNabola A, Rong H, et al. BAY 43-9006 exhibits broad spectrum oral antitumor activity and targets the RAF/MEK/ERK pathway and receptor tyrosine kinases involved in tumor progression and angiogenesis. Cancer Res 2004;64:7099-109.

8. Lim HY et al, Ann Surg Oncol 20(12):3747-53

9. Wang K, Lim HY, Shi S, Lee J, Deng S, Xie T, Zhu Z, Wang Y, Pocalyko D, Yang WJ, Rejto PA, Mao M, Park CK, Xu J. Genomic landscape of copy number aberrations enables the identification of oncogenic drivers in hepatocellular carcinoma. Hepatology. 2013 Aug;58(2):706-17

10. Kan et al, Whole-genome sequencing identifies recurrent mutations in hepatocellular carcinoma, Genome Res 2013

11 . Sung et al, Genome-wide survey of recurrent HBV integration in hepatocellular carcinoma, Nature Gen, 2012

12. Shankaran et al, Correlation of gene expression signatures and clinical outcomes in patients with advanced gastric cancer treated with pembrolizumab (MK-3475).

13. R. Cristescu, JY Lee et al Molecular analysis of gastric cancer identifies subtypes associated with distinct clinical outcomes, Nature Medicine 21, 449–456 (2015)

14. Gao Q, Wang XY, Qiu SJ, et al. Overexpression of PD-L1 significantly associates with tumor aggressiveness and postoperative recurrence in human hepatocellular carcinoma. Clin Cancer Res. 2009;15: 971-979.

15. Wang BJ, Bao JJ, Wang JZ, et al. Immunostaining of PD-1/PD-Ls in liver tissues of patients with hepatitis and hepatocellular carcinoma. World J Gastroenterol. 2011;17:3322-3329.

16. Budhu A, Forgues M, Ye QH, et al. Prediction of venous metastases, recurrence, and prognosis in hepatocellular carcinoma based on a unique immune response signature of the liver microenvironment. Cancer Cell. 2006;10:99-111.

18.0 APPENDICES

18.1 ECOG Performance Status

Grade	Description
0	Normal activity. Fully active, able to carry on all pre-disease performance without restriction.
1	Symptoms, but ambulatory. Restricted in physically strenuous activity, but ambulatory and able to carry out work of a light or sedentary nature (e.g., light housework, office work).
2	In bed <50% of the time. Ambulatory and capable of all self-care, but unable to carry out any work activities. Up and about more than 50% of waking hours.
3	In bed >50% of the time. Capable of only limited self-care, confined to bed or chair more than 50% of waking hours.
4	100% bedridden. Completely disabled. Cannot carry on any self-care. Totally confined to bed or chair.
5	Dead.

* As published in Am. J. Clin. Oncol.: *Oken, M.M., Creech, R.H., Tormey, D.C., Horton, J., Davis, T.E., McFadden, E.T., Carbone, P.P.: Toxicity And Response Criteria Of The Eastern Cooperative Oncology Group. Am J Clin Oncol 5:649-655, 1982.* The Eastern Cooperative Oncology Group, Robert Comis M.D., Group Chair.

18.2 Common Terminology Criteria for Adverse Events V4.0 (CTCAE)

The descriptions and grading scales found in the revised NCI Common Terminology Criteria for Adverse Events (CTCAE) version 4.0 will be utilized for adverse event reporting. (http://ctep.cancer.gov/reporting/ctc.html)

18.3 Response Evaluation Criteria in Solid Tumors mRECIST Criteria for Evaluating Response in Solid Tumors

Complete response (CR) is the disappearance of any all target lesions;
Partial response (PR) is at least a 30% decrease in the sum of diameters of viable target lesions, taking as reference the baseline sum of the diameters of target lesions;
Progressive disease (PD) is an increase of at least 20% in the sum of the diameters of viable target lesions, taking as reference the smallest sum of the diameters of viable target lesions recorded since the treatment started;
Stable disease (SD) is any cases that do not qualify for either partial response or progressive disease.
Non-target lesions:
CR is disappearance of all non-target lesions
SD is persistence of one or more non-target lesions PD is appearance of one or more new lesions and/or unequivocal progression of existing non-target lesions
mRECIST recommendations:
Pleural effusion and ascites: Cytopathologic confirmation of the neoplastic nature of any effusion that appears or worsens during treatment is required to declare PD.
