# Supplementary material for: Hepatocellular carcinoma patients with high circulating cytotoxic T cells and intra-tumoral immune signature benefit from pembrolizumab: results from a single-arm phase 2 trial
Source: Genome Med. 2022 Jan 6;14:1. doi: 10.1186/s13073-021-00995-8 (PMC8734300; doi:10.1186/s13073-021-00995-8)
Supplement: Supplementary file 2 — Additional file 2: The protocol revision history. [file 13073_2021_995_MOESM2_ESM.pdf]

Protocol Revision History

Ver. 3.3.1 (18 Jul 2019)

Ver. 3.3 (14 Dec 2018)

Ver. 3.2 (26 Jul 2018)

Ver. 3.1 (06 Dec 2017)

Ver. 3.0 (10 Oct 2017)

Ver. 2.0 (30 May 2017)

Ver. 1.3 (25 Jan 2017)

Ver. 1.2 (21 Nov 2016)
