# Supplementary material for: Hepatocellular carcinoma patients with high circulating cytotoxic T cells and intra-tumoral immune signature benefit from pembrolizumab: results from a single-arm phase 2 trial
Source: Genome Med. 2022 Jan 6;14:1. doi: 10.1186/s13073-021-00995-8 (PMC8734300; doi:10.1186/s13073-021-00995-8)
Supplement: Supplementary file 4 — Additional file 4: Table S2. Clinicopathological features associated with response to pembrolizumab (p value by Fisher’s exact test). Table S3. Safety profiles. Fig. S1. Survival analysis. (A-B) PFS and OS of all patients (n = 60). (C-D) PFS and OS according to PR vs. SD/PD. (E-F) PFS and OS according to PD vs. PR/SD. Fig. S2. Prevalence of tumor-infiltrating lymphocytes (A) and PD-L1 expression level (B) based on CTNNB1 somatic mutation. Fig. S3. Summary of nonsynonymous mutations in 47 HCC patients. Fig. S4. Correlation between the expression of neutrophil gene markers and NLR (A). Expression level of neutrophil gene markers according to NLR (B). Fig. S5. GSEA plots representing CHIANG_LIVER_CANCER_SUBCLASS_PROLIFERATION_UP pathway (A) and CHIANG_LIVER_CANCER_SUBCLASS_CTNNB1_DN pathway (B) were enriched in responders (PR). Patients with CTNNB1 mutation showed the higher expression levels of CTNNB1_UP geneset (C) and the lower expression levels of CTNNB1_DN geneset (D) [file 13073_2021_995_MOESM4_ESM.docx]

**Supplementary Table 2.** Clinicopathological features associated with response to pembrolizumab (*p* value by Fisher’s exact test)

|  | | PR | SD or PD | *p* value | PR or SD | PD | *p* value |
| --- | --- | --- | --- | --- | --- | --- | --- |
| **Age** | < 60  ≧ 60 | 1/25  5/28 | 24/25  23/28 | 0.196 | 9/25  7/28 | 16/25  21/28 | 0.284 |
| **Sex** | Female  Male | 4/12  2/41 | 8/12  39/41 | 0.019^*^ | 5/12  11/41 | 7/12  30/41 | 0.261 |
| **ECOG** | 0  1 | 1/8  5/45 | 7/8  40/45 | 1.00 | 2/8  14/45 | 6/8  31/45 | 0.544 |
| **AFP** | Low (< 400)  High (≧ 400) | 3/27  3/26 | 24/27  23/26 | 1.00 | 3/27  13/26 | 24/27  13/26 | 0.002 * |
| **PIVKA-II** | Low (<median)  High (≧median) | 4/27  2/26 | 23/27  24/26 | 0.669 | 5/28  11/25 | 23/28  14/25 | 0.038 * |
| **NLR** | Low (< 4)  High (≧ 4) | 6/29  0/24 | 23/29  24/24 | 0.027^*^ | 9/29  7/24 | 20/29  17/24 | 0.562 |
| **ALC** | Low (<median)  High (≧median) | 1/26  5/25 | 25/26  22/27 | 0.192 | 9/28  7/25 | 19/28  18/25 | 0.490 |
| **Extrahepatic**  **disease** | No  Yes | 0/6  6/47 | 6/6  41/47 | 1.00 | 2/6  14/47 | 4/6  33/47 | 0.595 |
| **Pre-CTx line** | 1  ≧ 2 | 5/42  1/10 | 37/42  10/11 | 1.00 | 12/42  4/11 | 30/42  7/11 | 0.436 |
| **Pre-RTx** | No  Yes | 3/25  3/28 | 22/25  25/28 | 1.00 | 5/25  11/28 | 20/25  17/28 | 0.109 |
| **CPS ≧ 1** | Negative  Positive | 0/21  4/19 | 21/21  15/19 | 0.042^*^ | 7/21  5/19 | 14/21  14/19 | 0.446 |

PR, partial response; SD, stable disease; PD, progressive disease; ECOG, Eastern Cooperative Oncology Group; AFP, alpha-feto protein; PIVKA-II, protein induced by vitamin K absence-II; NLR, neutrophile to lymphocyte ratio; ALC, absolute lymphocyte count; CTx, chemotherapy; RTx, radiotherapy; CPS, combine positive score.

**Supplementary Table 3.** Safety profiles

|  | Grade 1-2 | Grade 3-4 |
| --- | --- | --- |
| Fatigue | 8 (13.3%) | 0 |
| Prutitis | 6 (10.0%) | 0 |
| Skin rash | 5 (8.3%) | 0 |
| Diarrhea | 4 (6.7%) | 0 |
| Cough | 4 (6.7%) | 0 |
| Anorexia | 3 (5.0%) | 0 |
| Insomnia | 3 (5.0%) | 0 |
| Vertigo | 2 (3.3%) | 0 |
| Constipation | 2 (3.3%) | 0 |
| Hair loss | 1 (1.7%) | 0 |
| Hypersensitivity reaction | 0 | 1 (1.7%) |
| Thromboembolism | 0 | 1 (1.7%) |
| Elevated liver enzyme | 6 (10.0%) | 5 (8.3%) |
| Hyperbilirubinemia | 3 (5.0%) | 2 (3.3%) |
| Hypothyroidism | 1 (1.7%) | 1 (1.7%) |
| Hepatic encephalopathy | 0 | 1 (1.7%) |

**Supplementary Figure 1**. Survival analysis. (A-B) PFS and OS of all patients (n = 60). (C-D) PFS and OS according to PR vs. SD/PD. (E-F) PFS and OS according to PD vs. PR/SD.


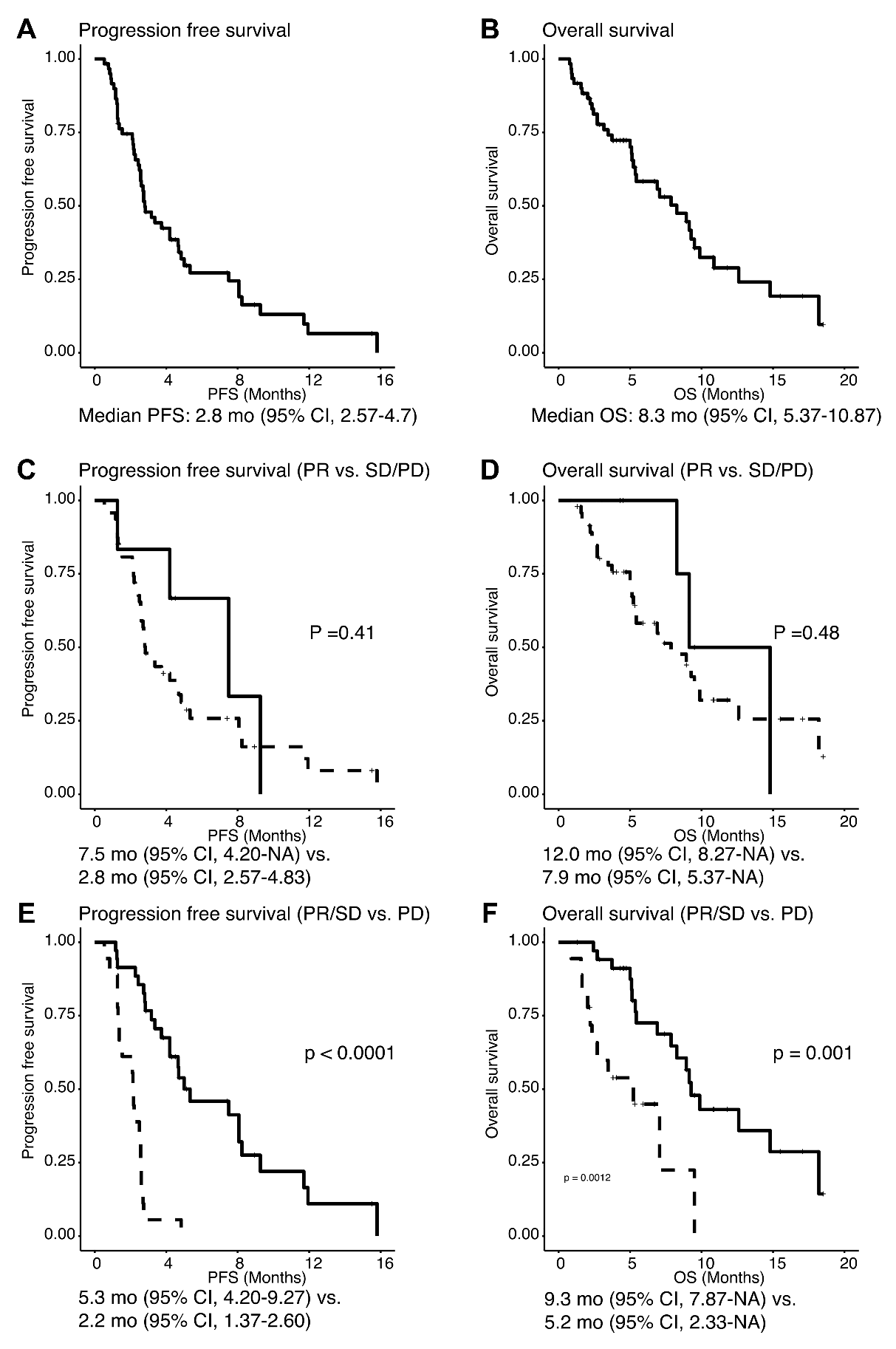


**Supplementary Figure 2.** Prevalence of tumor-infiltrating lymphocytes (A) and PD-L1 expression level (B) based on *CTNNB1* somatic mutation


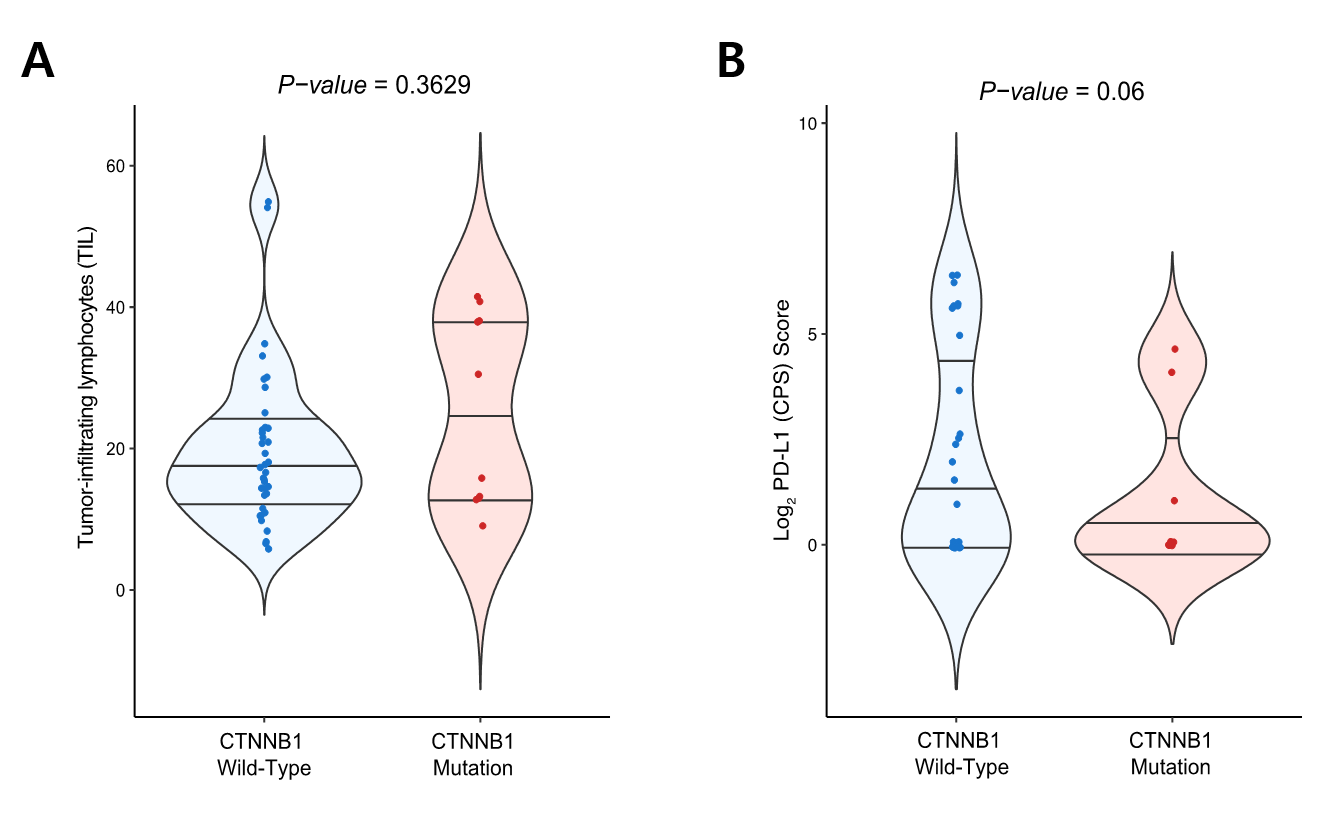


**Supplementary Figure 3.** Summary of nonsynonymous mutations in 47 HCC patients

**
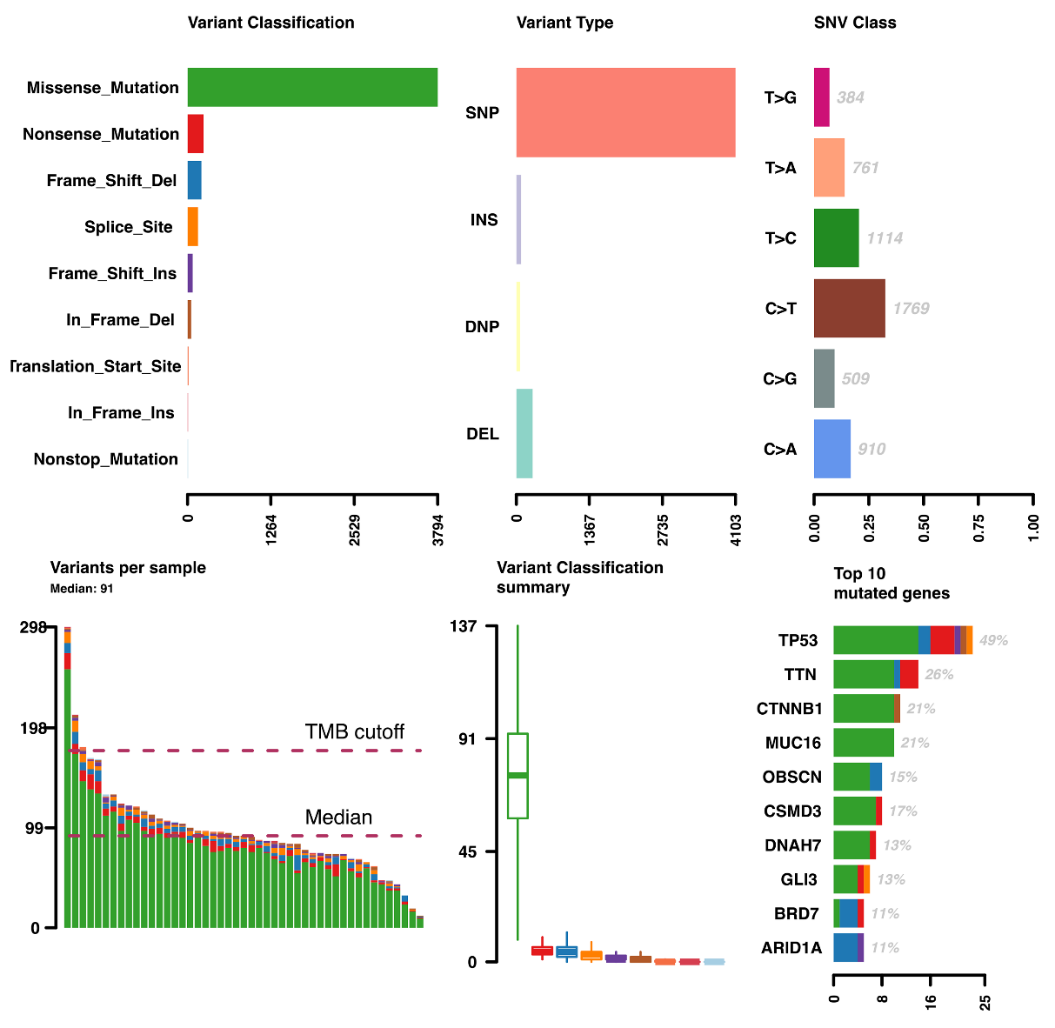
**

**Supplementary Figure 4.** Correlation between the expression of neutrophil gene markers and NLR (A). Expression level of neutrophil gene markers according to NLR (B)


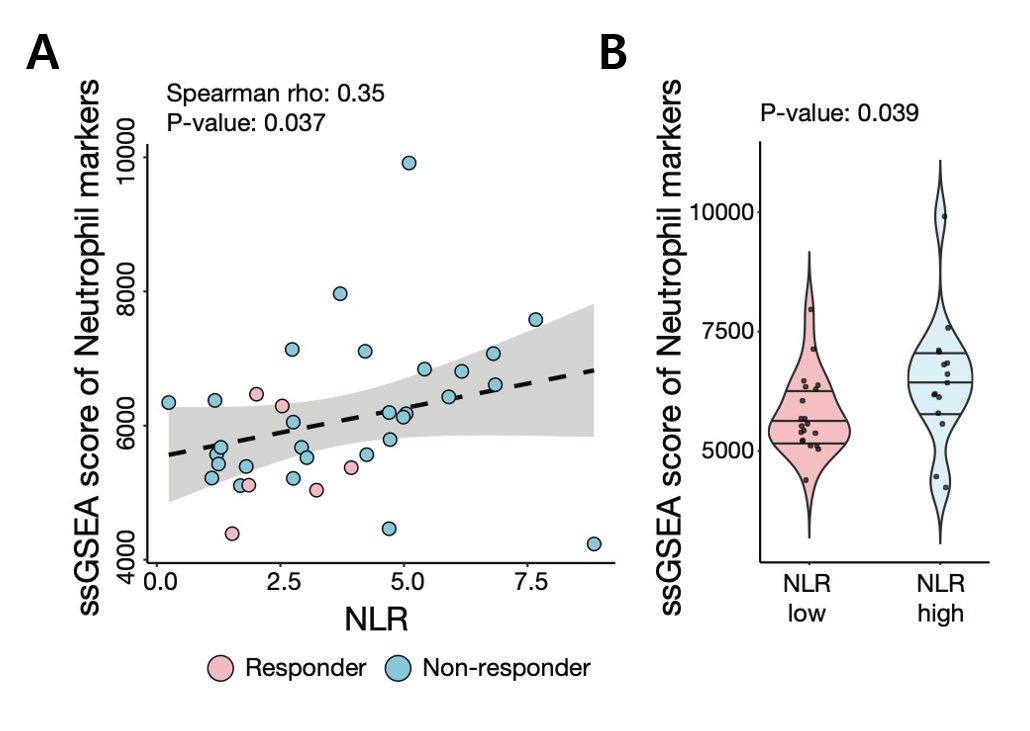


**Supplementary Figure 5.** GSEA plots representing CHIANG_LIVER_CANCER_SUBCLASS_PROLIFERATION_UP pathway (A) and CHIANG_LIVER_CANCER_SUBCLASS_CTNNB1_DN pathway (B) were enriched in responders (PR). Patients with CTNNB1 mutation showed the higher expression levels of CTNNB1_UP geneset (C) and the lower expression levels of CTNNB1_DN geneset (D)

**
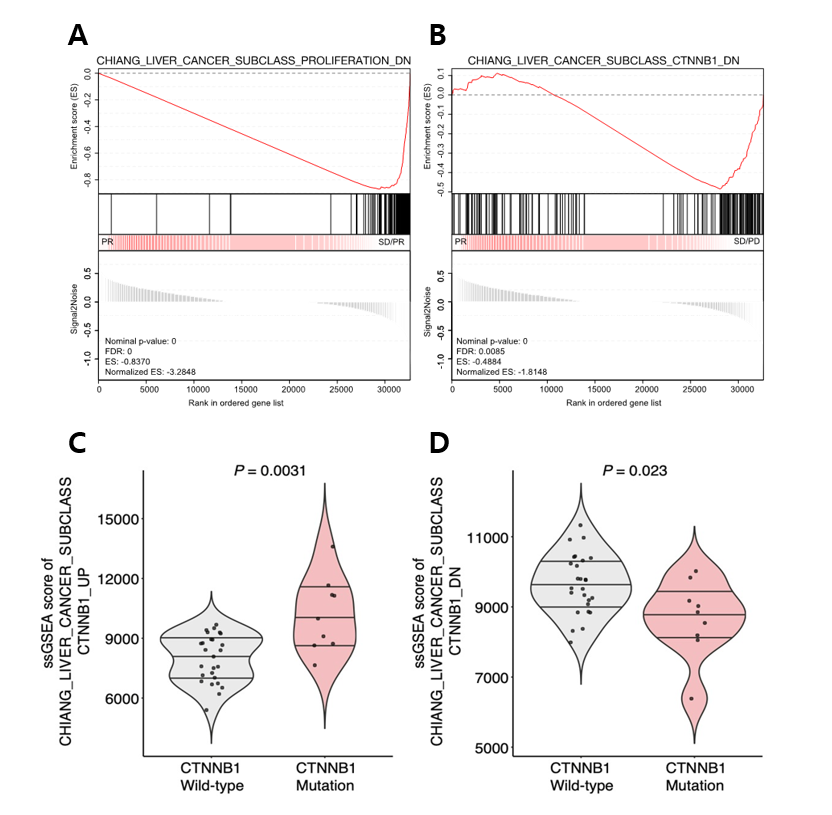
**
